# Haplotype-resolved genome assemblies of BJ and IMR-90 human fibroblast cell lines reveal extensive structural variation and enable reanalysis of historical sequencing data

**DOI:** 10.1093/nar/gkag333

**Published:** 2026-04-29

**Authors:** T Rhyker Ranallo-Benavidez, Yue Hao, Emilia Volpe, Maryam Jehangir, Noelle Fukushima, Zachary D Stephens, Ogechukwu Mbegbu, Matteo T Ungaro, Rebecca Reiman, Jessica Molnar, Danyael Murphy, Dorothy Marie Paredes, Shukmei Wong, Kara Karaniuk, Stephanie Buchholtz, Jean-Pierre Kocher, Jonathan Keats, Mitchell J Machiela, Mikhail Kolmogorov, Benedict Paten, Simona Giunta, Floris P Barthel

**Affiliations:** Translational Genomics Research Institute, Phoenix, AZ, 85004, United States; Translational Genomics Research Institute, Phoenix, AZ, 85004, United States; Department of Biology and Biotechnology “Charles Darwin”, Sapienza University, Rome, 00185, Italy; Translational Genomics Research Institute, Phoenix, AZ, 85004, United States; Translational Genomics Research Institute, Phoenix, AZ, 85004, United States; Quantitative Health Sciences, Mayo Clinic, Rochester, MN, 55905, United States; Translational Genomics Research Institute, Phoenix, AZ, 85004, United States; Department of Biology and Biotechnology “Charles Darwin”, Sapienza University, Rome, 00185, Italy; Translational Genomics Research Institute, Phoenix, AZ, 85004, United States; Translational Genomics Research Institute, Phoenix, AZ, 85004, United States; Translational Genomics Research Institute, Phoenix, AZ, 85004, United States; Translational Genomics Research Institute, Phoenix, AZ, 85004, United States; Translational Genomics Research Institute, Phoenix, AZ, 85004, United States; Translational Genomics Research Institute, Phoenix, AZ, 85004, United States; Translational Genomics Research Institute, Phoenix, AZ, 85004, United States; Quantitative Health Sciences, Mayo Clinic, Rochester, MN, 55905, United States; Translational Genomics Research Institute, Phoenix, AZ, 85004, United States; Division of Cancer Epidemiology and Genetics, National Cancer Institute, Rockville, MD, 20850, United States; Center for Cancer Research, National Cancer Institute, NIH, Bethesda, MD, 20892, United States; UC Santa Cruz Genomics Institute, University of California, Santa Cruz, CA, 95060, United States; Department of Biology and Biotechnology “Charles Darwin”, Sapienza University, Rome, 00185, Italy; Translational Genomics Research Institute, Phoenix, AZ, 85004, United States

## Abstract

We present chromosome-level, phased diploid genome assemblies of two widely used human fibroblast cell lines: BJ (46,XY) and IMR-90 (46,XX). Using Oxford Nanopore, PacBio HiFi, and Hi-C sequencing data, we generated assemblies spanning 5.9 and 6.0 Gbp with diploid quality values exceeding QV 60. To validate structural integrity, we developed KaryoScope, an alignment-free tool for generating computational karyograms from *k*-mer feature databases. We identify >50 000 structural variants relative to T2T-CHM13v2.0, the majority of which are heterozygous and cell-line-specific. Combining reference-based and *de novo* gene annotation, we uncover a previously unreported 1 Mbp homozygous duplication at the 16p11.2 locus in BJ, demonstrating that even karyotypically normal cell lines can harbor clinically relevant submicroscopic rearrangements. We show that mapping publicly available short-read, RNA-seq, and ChIP-seq data to sample-matched diploid assemblies substantially improves read alignment and enables haplotype phasing of 23%–28% of short reads. The BJ and IMR-90 assemblies and associated variant calls are publicly available as a resource for the research community.

## Graphical Abstract

**Graphical Abstract:**
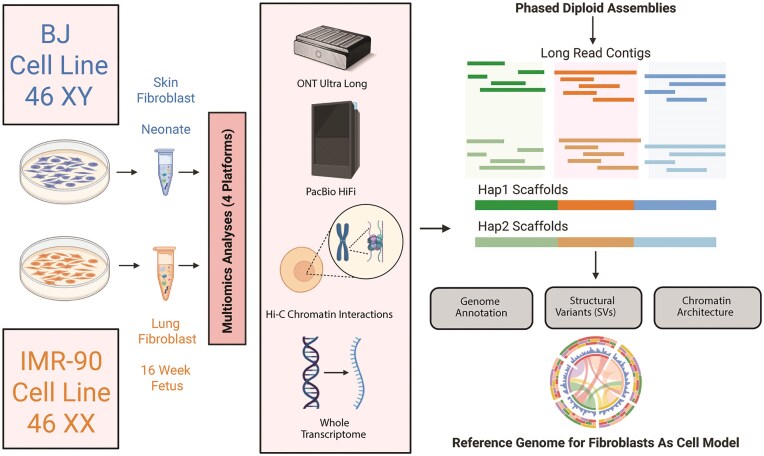

## Introduction

Human cell lines are essential tools driving innovation in biomedical research. Their adaptation to *in vitro* culture conditions allows them to be used as robust model systems to study response to diverse experimental conditions, understand fundamental cellular processes, and elucidate disease mechanisms [1]. Two widely utilized cell lines are the finite BJ and IMR-90 human fibroblast lines, which exhibit a stable diploid karyotype over many population doublings but enter permanent growth arrest (senescence) after a fixed number of passages [2, 3]. The BJ cell line (46,XY), derived from neonatal foreskin tissue, has been used to study telomere shortening [4], the derivation of stem cell lines [5], chromatin accessibility [6], and the premature aging disease Hutchinson–Gilford progeria syndrome [7]. The IMR-90 cell line (46,XX), derived from fetal lung tissue, has been used to study cell motility [8], viral susceptibility [3], senescence [9], and chromosome instability in cancer progression [10].

Despite having been used in research for several decades, the complete genetic background of these foundational cell lines remains largely unexplored at a haplotype-resolved level. The accurate interpretation of biological experimentation in cell lines requires a comprehensive understanding of their genetic context. While reference genomes such as GRCh38 [11] and, more recently, T2T-CHM13v2.0 [12] provide a valuable framework, they do not capture the extensive genomic diversity present across individuals and, importantly, within laboratory cell lines. Recognizing this limitation, the Human Pangenome Reference Consortium (HPRC) aims to create a more complete and diverse human pangenome reference by assembling high-quality, phased genome sequences from hundreds of individuals representing global genetic diversity [13]. The HPRC has made significant strides in generating highly contiguous, telomere-to-telomere assemblies, revealing previously uncharacterized structural variation and improving the representation of complex genomic regions [14].

The current study complements and extends the critical work of the HPRC by focusing on the *de novo* assembly of diploid genomes of the BJ and IMR-90 cell lines. While the HPRC focuses on assembling genomes from diverse human individuals, our work addresses a distinct, yet equally important, need: characterizing the specific genomic landscapes of widely used *in vitro* model systems. Cell lines, through their derivation and propagation over time, are thought to accumulate substantial genomic alterations, including single nucleotide variants (SNVs), insertions and deletions (indels), and, crucially, large-scale structural variants (SVs) that can significantly impact cellular phenotypes and experimental outcomes [15]. These alterations are often unique to a specific cell line and are not represented in population-based pangenome references.

Here, we present publicly available chromosome-level assemblies of the BJ and IMR-90 cell lines. Importantly, the assemblies are diploid, meaning that both parental haplotypes are resolved for each cell line. Haplotype resolution is essential for accurately representing and phasing genetic variants, identifying allele-specific gene expression, and understanding the functional consequences of structural variation [16]. Many previous genomic studies have relied on mapping to the reference genome, which can lead to biases and incomplete or inaccurate variant calling, particularly in regions of high divergence or structural complexity [17]. These *de novo* diploid assemblies overcome those limitations. We show that the BJ and IMR-90 genomes harbor >50 000 SVs relative to T2T-CHM13v2.0, the majority of which are heterozygous and cell-line-specific. Combining reference-based and *de novo* gene annotation, we uncover a previously unreported homozygous duplication at 16p11.2 in BJ. We further demonstrate that mapping historical short-read, RNA-seq, and ChIP-seq data to the matched haplotype-resolved assemblies substantially improves alignment quality and enables straightforward haplotype phasing. By providing chromosome-level, phased genomes for BJ and IMR-90, we enable more accurate and reproducible research using these cell lines and their derivatives.

## Materials and methods

### Ethics statement

A central consideration for this study was to ensure that we were free to publicly release the BJ and IMR-90 assemblies. There are ethical considerations around the public release of the data because these commercially purchased cell lines were derived before formal research use consent for biospecimens collected for clinical purposes was widely adopted. At that time, patient-derived biological materials were often used in research without explicit consent for future applications. This is likely the case for these lines, therefore WCG IRB was asked to review the proposal to share the data. The IRB assessed the risks of identifying the original subjects or their immediate family members, as well as the potential risks to groups or populations associated with submitting data to NIH-designated repositories and its subsequent sharing. Critically, openly releasing this data will significantly benefit many researchers using BJ, IMR-90, or derivative models in their laboratories, including studies of basic cell biology, disease modeling, and drug development.

Importantly, both BJ and IMR-90 are part of the National Institutes of Health (NIH) Common Fund 4D Nucleome Project and the National Human Genome Research Institute ENCODE Project. Large amounts of sequencing data on these cell lines are currently already publicly available through these consortia. After presenting all this information to the IRB, it was determined that the benefits of sharing the data publicly outweigh the risks and we were permitted to release our results.

### Cell lines

BJ (ATCC CRL-2522) and IMR-90 (ATCC CCL-186) fibroblasts were received from Michael Berens and Haiyong Han at the Translational Genomics Research Institute. They were cultured in Eagle’s Minimum Essential Medium (ATCC, Cat. No. 30-2003) +10% FBS (VWR, Cat. No. 97068-091) according to ATCC subculturing procedures. Both cell lines were authenticated by STR profiling at ATCC (https://www.atcc.org/, last accessed 27 February 2026). Cells for sequencing were harvested with 0.25% Trypsin–ethylenediaminetetraacetic acid (EDTA) (Thermo Fisher, Cat. No. 25200072), washed with 1× PBS (pH 7.4; Thermo Fisher, Cat. No. 10010023), counted on a Countess™ II FL Automated Cell Counter (Thermo Fisher, Cat. No. AMQAF1000), and used as input for NEB Monarch HMW genomic DNA (gDNA) isolation, Hi-C crosslinking, RNeasy Plus Mini isolation, or frozen as dry pellets and stored at −80°C for use in Oxford Nanopore Technologies (ONT) Ultra-Long (UL) library preparations. Estimated cumulative population doubling level (PDL) calculations were performed using PDL = 3.32 (logXe-logXb) + S where log is the base 10 logarithm, Xb is the cell number at the beginning of the incubation period, Xe is the cell number at the end of the incubation period, and S is the starting PDL (ref ATCC Animal Cell Culture Guide ACCG-042024-v08, p6).

### gDNA isolation and QC

BJ and IMR-90 cells were used as input for gDNA isolation using the NEB Monarch HMW DNA extraction kit for cells and blood (New England Biolabs, Cat. No. T3050L) following manufacturer’s instructions. HMW DNA was quantified by Nanodrop and Qubit™ 1xdsDNA High Sensitivity kit (Thermo Fisher, Cat. No. Q33231). Rough size distribution and DNA integrity number were measured with Genomic DNA ScreenTape and reagents (Agilent, Cat. Nos 5067-5365 and 5067-5366) using the Agilent 4150 TapeStation System (Agilent, Cat. No. G2992AA).

### PacBio HiFi library preparation and sequencing

Two to five micrograms of HMW gDNA was used as input for SMRTbell Prep Kit V3.0 (PacBio, Cat. No. 102-182-700). Mechanical shearing of gDNA was performed with Megaruptor 3 (Diagenode, Cat. No. B06010003) using the standard protocol with target size of 15–20 kb. Fragmentation was analyzed by Femto Pulse System (Agilent, Cat. No. M5330AA). SMRTbell libraries were sequenced on the PacBio Revio platform with SMRT Cell tray (PacBio, Cat. No. 102-202-200), loaded at 240–260 pM, with 30-h movie collection times. For BJ, two libraries were made and run on 3 flowcells to reach >30× coverage of the genome. For IMR-90, two libraries were made and run on 2 flowcells to reach >30× coverage of the genome.

### Hi-C crosslinking

1–3 × 10^6^ freshly harvested cells were crosslinked using 1% methanol-free formaldehyde (Pierce, Cat. No. 28 906) in 1× phosphate-buffered saline (PBS) (pH 7.4) for 10 min with gentle agitation on a platform rocker. Crosslinking reactions were quenched with 200 mM Glycine at RT 5 min with rotation at 10 RPM followed by 15 min incubation on ice. Crosslinked cells were washed in 1× PBS (pH 7.4), frozen as dry pellets, and stored at −80°C.

### Hi-C library preparation and sequencing

Hi-C assays were performed on frozen crosslinked cell pellets using the Arima High Coverage HiC Kit (Arima Genomics, Cat. No. A101030) and libraries were prepared using Arima Library Prep Kit v2 (Arima Genomics, Cat. No. A303011) per manufacturer’s instructions. 1.5 µg of proximally ligated DNA suspended in 130 µl of the elution buffer was added to a microTUBE (Covaris, Cat. No. 520 045) and sonicated using a Covaris E220 with the following parameters: temperature (4°C–7°C), peak incident power (105), duty factor (5%), cycles per burst (200), treatment time (75 s). Post-sonication QC was performed on a TapeStation HSD5000 (Agilent Technologies, Cat. No. 5067–5592) to verify that fragment sizes were between 550 and 600 bp prior to size selection. Size selection was performed with AMPure XP beads (Beckman Coulter, Cat. No. A63881) to collect fragments >400 bp. Two hundres nanograms of size-selected DNA was put into biotin enrichment. The enriched products were end-repaired, adapter-ligated, and tagged with manufacturer supplied P7 and P5 8 bp index pairs using 5 cycles of polymerase chain reaction (PCR). The final bead-purified libraries were quantified by Qubit dsDNA HS (Thermo Fisher, Cat. No. Q33231) and TapeStation HSD5000 screentape, pooled, and re-quantified. The pool was sequenced on a NovaSeqX 10B flow cell (Illumina, Cat. No. 20 085 594) for 151 × 151 cycles with a 1% PhiX spike-in to 25× coverage.

### Oxford nanopore technologies ultra-long sequencing

Dry cell pellets of 6 × 10^6^ of each cell type were used as input for ONT-UL library preparation per Ultra-Long DNA Sequencing Kit V14 (Oxford Nanopore Technologies, Cat. No. SQK-ULK114). Libraries were loaded on PromethION Flow Cells (R10.4.1) (Oxford Nanopore Technologies, Cat. No. FLO-PRO114M). BJ and IMR-90 libraries were loaded onto 7 and 9 flowcells, respectively, to reach >20× coverage of the genome for each cell line.

### RNA isolation and QC

For each cell pellet, total RNA was isolated with RNeasy Plus Mini (Qiagen, Cat. No. 74134) using QIAshredder columns (Qiagen, Cat. No. 79654) for homogenization, then purified with Zymo RNA Clean & Concentrator-5 (Zymo Research, Cat. No. R1015). Isolated RNA was measured for quantity with Quant-iT Ribogreen RNA Assay (Thermo Fisher, Cat. No. R11490) and quality with High Sensitivity RNA ScreenTape and buffer (Agilent, Cat. No. 5067–5579 and 5067–5580).

### Whole transcriptome library preparation and sequencing

For each RNA sample, a uniquely dual-indexed, Illumina-compatible, double-stranded complementary DNA whole transcriptome library was synthesized from 1 µg of total RNA with Takara Bio’s SMARTer Stranded Total RNA Sample Prep Kit—HI Mammalian kit (Takara Bio, Cat. No. 634874) and SMARTer RNA Unique Dual Index kit (Takara Bio, Cat. No. 634452). Briefly, this library preparation included ribosomal depletion, RNA fragmentation (94°C for 3 min), and a 12-cycle indexing and enrichment PCR. Each library was measured for size with Agilent’s High Sensitivity D1000 ScreenTape and buffer (Agilent, Cat. No. 5067–5584 and 5067–5603). One microliter of each library was combined into a nonequimolar pool that was then measured for size with TapeStation and concentration with Roche’s KAPA SYBR FAST Universal qPCR Kit (Roche, Cat. No. KK4824), diluted to 70 pM, then loaded into an iSeq flow cell cartridge (Illumina, Cat. No. 20031371) with a 1% (v/v) PhiX Control v3 spike-in (Illumina, Cat. No. FC-110-3001), and sequenced to 101 × 9 × 9 × 101 cycles. Passing filter cluster counts per library from these iSeq data were used to make a rebalanced pool that was subsequently measured for size and concentration, diluted to 750 pM with a 1% (v/v) PhiX Control v3 spike-in, denatured and further diluted, loaded into a NovaSeq X flow cell lane (Illumina, Cat. No. 20085594), and sequenced at 151 × 8 × 8 × 101 cycles with a final flow cell concentration of 150 pM. Libraries were sequenced to at least 50 M read pairs (or 100 M paired-end reads). The low diversity of the first three bases in Read 1 of these libraries was balanced in both the iSeq and NovaSeq X pools with other library types that had a higher diversity across those bases; otherwise, a 20% PhiX spike-in would have been needed for both pools.

### Multicolor fluorescence *in situ* hybridization 

BJ and IMR-90 cells were cultured to 70% confluency, treated with 0.1µg/ml Colcemid solution (Thermo Fisher, Cat. No 15212012) to arrest cells in metaphase, and harvested with 0.5% Trypsin–EDTA (Gibco, Cat. No. 15400054). Cells were pelleted by centrifugation at 1500 rpm for 5 min, resuspended with 0.075 M KCl (Invitrogen, Cat. No. AM9640G) for 10 min, then fixed with ice-cold Methanol:Acetic Acid solution at a 3:1 ratio, respectively. Thirty microliters of cell suspension was dropped onto pre-cleaned slides and hybridized to human multicolor fluorescence *in situ* hybridization (M-FISH) probe (MetaSystems, Cat. No. D-0125-060-DI) following the manufacturer’s protocol. Coverslips were mounted on slides using ProLong Glass Antifade with NucBlue (Thermo Fisher, Cat. No. P36981). Images were acquired using a Zeiss AxioImager Z2 equipped with the MetaSystems Metafer Slide Scanning Platform (MetaSystems, Metafer5).

### M-FISH karyogram analysis

Karyograms were generated with the Interactive Karyotyping system (Ikaros) module, a feature of the Metafer system. Captured images were processed by background subtraction, object thresholding, object separation, and pixel calling. False colors were assigned to each chromosome for clear identification. Chromosome assignments were confirmed by comparing fluorescent spectra to the manufacturer labeling scheme for each chromosome. PDF reports for each cell line were generated and exported using MetaSystems’ Neon data management software.

### Sequencing QC

HiFi read ubam files were converted to fasta using Samtools (version 1.21) [18]. ONT-UL reads were basecalled using Dorado (version 0.7.2 + 9ac85c6, https://github.com/nanoporetech/dorado, last accessed 27 February 2026) with the dna_r10.4.1_e8.2_400bps_sup@v5.0.0 model to produce duplex ubam files. The ubams were converted to fastq using Samtools (version 1.21). We retained the reads with the dx tag set to 1 or 0 to keep the duplex reads and the simplex reads without duplex offsprings. The first five bases from the 5′ end of both read 1 and read 2 of the Hi-C data were trimmed using cutadapt (version 4.5) [19].

We constructed *k*-mer histograms for the long-read sequencing data using kmc (version 3.2.4) [20]. We analyzed the *k*-mer histograms using GenomeScope (version 2.0.1) [21] to estimate the coverage, heterozygosity, and error rate of the data. We used NanoPlot (version 1.44.0) [22] to generate read length versus average read quality KDE plots and get the total sequencing coverage, read length N50, and median quality scores of the sequencing data. We generated coverage versus read length plots using a python script. We aligned the Hi-C data to T2T-CHM13v2.0 [12] with bowtie2 (version 2.5.4) [23] and used hic_qc (version 1.0, https://github.com/phasegenomics/hic_qc, last accessed 27 February 2026) to plot the distribution of long range contacts.

### Genome assembly

We generated phased diploid assemblies of the cell lines using Verkko (version 2.2.1) [24]. Prior to assembly, the ONT-UL data were error-corrected using Dorado (version 0.8.1 + c3a2952). The HiFi reads and the error corrected ONT-UL reads were given as hifi inputs to Verkko. The ONT-UL reads and the Hi-C reads were given as nanopore and hic inputs, respectively, to Verkko.

### Mitochondrial genome assembly

The mitochondrial genome was not included in the chromosomal assemblies, as Verkko assembles nuclear chromosomes and does not reconstruct organellar genomes. Complete mitochondrial sequences were identified among Verkko’s unassigned contigs as concatenated multimeric copies, consistent with the high copy number of mtDNA. A single-copy mitochondrial genome was extracted from each cell line, yielding sequences of ~16 568 bp with ≥99.7% identity to the revised Cambridge Reference Sequence (rCRS; NC_012920.1) and 100% query coverage.

### Assembly curation

Hi-C reads were aligned to the pre-curation diploid assemblies (including hap1, hap2, and the unassigned contigs) and processed according to the Arima Mapping pipeline (A160156 v03, https://github.com/ArimaGenomics/mapping_pipeline, last accessed 27 February 2026). In summary, Hi-C reads were aligned to the pre-curation diploid assemblies using BWA mem with default parameters (version 0.7.18-r1243-dirty) [25]. The bam files were filtered using the filter_five_end.pl script to retain only the portion of chimeric reads that map in the 5′-orientation. The two_read_bam_combiner.pl script was used to combine the single-end Hi-C reads. Picard (version 3.1.1, https://github.com/broadinstitute/picard, last accessed 27 February 2026) was used to add read groups to the bam files and remove optical duplicates. Hi-C contact maps were created with PretextMap (version 0.1.9, https://github.com/sanger-tol/PretextMap, last accessed 27 February 2026).

Using PretextView (version 0.2.5, https://github.com/sanger-tol/PretextView, last accessed 27 February 2026) for visualization of this contact map, we were able to detect misjoins and consolidate segments from the same chromosomes into a single scaffold. Based on this refined structure, we proceeded to generate an AGP (A Golden Path) file to define the scaffold organization. This file was subsequently used as input for the Rapid Curation 2.0 pipeline (https://github.com/Nadolina/Rapid-curation-2.0, last accessed 27 February 2026) to separate the two haplotypes [26].

### Assembly QC

Bandage (version 0.8.1) [27] was used to view the assembly graphs from Verkko. gfastats (version 1.3.9) [28] was used to calculate the genome size, total gap length, number of gaps, contig and scaffold NG50, and GC content of the assemblies. Because each haplotype assembly contains only a single sex chromosome, we used the total size of T2T-CHM13v2.0 excluding chrX (2 963 015 935 bp) as the expected genome size for BJ hap1 (which contains chrY) and the total size excluding chrY (3 054 815 472 bp) for the remaining three haplotypes (which contain chrX). NG50 is defined as the maximum sequence length such that all sequences of that length or longer together cover at least 50% of the reference genome size. LG50 is the corresponding number of sequences needed to reach this threshold. The NGx and LGx curves plot the entire range of NG and LG values. The auN value represents the area under the Nx curve and is independent of the expected genome size. The NGx and LGx charts were plotted using a custom R script. The auN values of the assembly were calculated using a custom Python script.

Compleasm (version 0.2.6) [29] with the primates lineage was used to evaluate genome completeness by searching for near-universal single-copy ortholog gene sequences [30]. Compleasm was run on each haplotype assembly as well as the single-sex-chromosome versions of T2T-CHM13v2.0 to provide matched baselines for comparison. Meryl (version 1.4.1) [31] was used to count the 31-mers from the HiFi data and publicly available Illumina data (see the “Data Availability” section) [32, 33]. The quality of the assemblies was evaluated using Merqury (version 1.3) [31].

The HiFi reads were aligned to the diploid assemblies using minimap2 (version 2.28-r1209) [34] and HMM-Flagger (version 1.0.0) [13] was used to detect any misassemblies. Liftoff (version 1.6.3) [35] was used to lift the annotation of T2T-CHM13v2.0 separately to each haplotype of the assemblies.

RepeatMasker (version 4.1.6, http://www.repeatmasker.org/, last accessed 27 February 2026) was used to profile the repeat sequences in the assemblies using the Dfam withRBRM (version 3.8) database [36] and the ABBlast search engine. The RepeatMasker script buildSummary.pl was used to produce the repeat summary stats. RepeatMasker defines simple repeats as micro-satellites, and low-complexity DNA as poly-purine or poly-pyrimidine stretches or regions of extremely high AT or GC content.

AlcoR (version 1.9) [37] was used to map and visualize low-complexity regions (LCRs) in the assemblies. For each cell line, both haplotype assemblies were concatenated into a single file, allowing the distant-range model to detect inter-haplotype as well as intra-chromosomal repetitive similarity. The AlcoR mapper was run three times per concatenated file, each time with a different context model memory limit to capture LCRs at distinct spatial scales: distant LCRs (context order 14, unlimited memory), medium-range LCRs (context order 13, memory limited to 50 000 symbols), and local LCRs (context order 13, memory limited to 5000 symbols). Each run used a compression threshold of 0.75, a smoothing window of 5000, and a minimum region size of 5000 bp (--ignore 5000). A distinct color hue was assigned to each distance class (blue for distant, green for medium range, red for local), and the resulting per-chromosome annotations were concatenated and visualized as haplotype-resolved maps.

### KaryoScope computational karyogram analysis

KaryoScope annotates DNA sequences in an alignment-free manner by querying them against *k*-mer feature databases in which the keys are short substrings of DNA (31 bp *k*-mers by default) and the values are genomic features of interest. For this paper, we built a database of the 31-mers in T2T-CHM13v2.0 with 50 distinct features (telomere, centromere, and the 48 chromosome-specific arms). For visualization, the telomere feature is assigned the color blue, centromere is assigned orange, and each of the chromosomes is assigned a distinct color. Within a given chromosome, the p arm and q arm are shaded light and dark, respectively. *K*-mers present in multiple features (e.g. present on multiple chromosomes) are assigned the color gray. Novel *k*-mers not present in the database represent sequence differences with respect to T2T-CHM13v2.0 and are assigned the color white. To generate the computational karyograms, KaryoScope draws each contig or scaffold of an assembly as a colored vertical rectangle to identify the positions of each feature within the sequence. By default, each pixel represents 250 Kbp of sequence and is colored according to majority rule (e.g. if the majority of *k*-mers in that region are centromere *k*-mers then the pixel is colored orange). The sequences are drawn in order of their corresponding chromosomes and haplotypes. Sequences smaller than 1 Mbp are filtered out and not drawn.

### Centromere analysis

Centromere annotation was done by intersecting the outputs derived from RepeatMasker and HumAS-HMMER for AnVIL (https://github.com/fedorrik/HumAS-HMMER_for_AnVIL, last accessed 27 February 2026) to identify the position of the centromeres and the higher-order repeats (HORs) and superfamilies (SF) within each centromere. RepeatMasker was run with default parameters, while HumAS-HMMER for AnVIL was applied as described in [38]. To generate heatmaps showing the variation between centromeres, StainedGlass (version 6.7.0) [39] was run with the following parameters: window = 5000 and mm_f = 30000. Monomer-by-monomer annotation and SVs detection within the live HORs was done using StV [38]. Centeny maps and centromere architecture annotations using the Genomic Centromere Profiling (GCP) pipeline Model 1 and Model 2 were generated as previously shown [40].

### Telomere analysis on assemblies and sequencing reads

Assembly-based telomere quantification was performed on the four assembled haplotypes using seqtk telo (version 1.5-r133, https://github.com/lh3/seqtk, last accessed 27 February 2026). Read-based telomere quantification was performed for BJ and IMR-90 by applying telogator2 (version 2.2.3) [41] to the long-read sequencing data (HiFi and ONT combined). Assembly-based telomere quantification was performed on all samples of release 2 of the HPRC (*n* = 231) using seqtk telo (Supplementary Table S4). We performed read-based telomere quantification on a subset of these (*n* = 49) using telogator2 (Supplementary Table S5). We fit a linear regression, and the Pearson correlation coefficient was calculated to assess the linear relationship between assembly-based telomere lengths and read-based estimates (Supplementary Fig. S7C).

### Microbial contaminant screening

The Foreign Contamination Screening (FCS) tool (version 0.5.4) [42] from NCBI was used to detect adaptor and vector contamination in the post-curation assemblies. FCS was also used to screen for nonhuman sequences in the post-curation assemblies. No contaminant sequences were identified in any of the four haplotype assemblies.

To screen for viral integration events, 12 viral reference genomes were downloaded from NCBI (Supplementary Table S6). Nucleotide BLAST databases were created for each post-curation haplotype assembly using makeblastdb (BLAST + version 2.17.0) [43]. BLASTn searches were performed with the following parameters: *e*-value 1*e*-5, -dust no, -task blastn. Hits were filtered at two thresholds: a relaxed threshold (≥80% identity, ≥100 bp alignment) and a stringent threshold (≥90% identity, ≥500 bp alignment). Stringent hits were intersected with telomere annotations to determine genomic context.

### Synteny analysis

NtSynt (version 1.0.2) [44] was used to produce the multisample synteny plot between T2T-CHM13v2.0 and the four assembled haplotypes. SVbyEye (version 0.99.0) [45] was used to produce the synteny plots of hap1 versus hap2 for BJ and IMR-90. The synteny plots of the IMR-90 haplotypes to T2T-CHM13v2.0 showing the chromosome 8 inversion were produced by SyRI (version 1.6.3) [46].

### Variant analysis

SyRI was used to call variants of each of the four assembly haplotypes against T2T-CHM13v2.0. SNVs and indels were compared against the gnomAD (version 4.1.0) [47] dataset. The resulting intersection data were reformatted into a binary matrix for UpSet plot analysis using ComplexUpset (version 1.3.3, https://krassowski.github.io/complex-upset/, last accessed 27 February 2026). Variants exclusive to gnomAD were excluded from the UpSet plot. SVs were merged across the haplotypes with Jasmine (version 1.1.5) [48], and SVs shorter than 50 bp were removed. The stacked upset plot of SV counts was generated using the UpSetPlot Python package (version 0.9.0, https://github.com/jnothman/UpSetPlot, last accessed 27 February 2026) [49]. Individual SVs were visualized using IGV (version 2.18.4) [50] and Ribbon (version 2.0) [51]. The Ribbon website (https://v2.genomeribbon.com/, last accessed 24 February 2026) can be used to visualize all reads from a given dataset spanning a target region. Three example SVs were selected to illustrate cell-line-specific structural changes in BJ and IMR-90. The target coordinates for these examples are chr2:78 850 000–78 860 000, chr5:75 600 000–75 610 000, and chr10:57 630 000–57 650 000. Target regions are shown as black boxes in the Ribbon visualizations, with insertion events shown as red dots and deletion events as gaps on each read.

To independently assess assembly-based SV calls, we performed alignment-based SV calling using Sniffles2 (version 2.4) [52]. For each sample, PacBio HiFi and Oxford Nanopore reads were aligned to T2T-CHM13v2.0 using minimap2 (with the map-hifi and map-ont presets), sorted with samtools, and processed through the Sniffles2 multisample calling workflow to produce a combined VCF per sample. The same reads were also aligned to each sample’s matched diploid assembly and processed identically. Assembly-based SV calls from SyRI were preprocessed to standardize type annotations and normalize allele representations. Only SVs ≥50 bp were considered.

### Ancestry analysis

For the ancestry analysis, RFMix2 tool (version 2) [53] was used to infer local ancestry for each haplotype of the assemblies. As a reference panel, the phased VCF from the 1000 Genomes Project [54] were downloaded. The target dataset consisted of phased variants generated using HiPhase (version 1.5.0-8af3f1e) [55] and DeepVariant (version 1.9.0) [56]. Local ancestry inference was performed using RFMix2 with default parameters. To visualize chromosome-level ancestry, the AncestryGrapher toolkit (version2) [57] was employed, which provides chromosome painting representations.

### Novel protein-coding gene analysis

To identify putative novel protein-coding genes in each haplotype, we applied a multifilter pipeline combining reference-based annotation with evidence-informed *de novo* gene prediction. Known genes were identified by lifting T2T-CHM13v2.0 reference annotations onto each haplotype using Liftoff (version 1.6.3), which aligns reference gene models to the target genome. Separately, *de novo* gene predictions were generated using BRAKER3 (version 3.0.8) [58], which integrates both genomic sequence features (e.g. splice site motifs) and RNA-seq evidence. Novel gene candidates were defined as BRAKER3-predicted genes with no overlap to any Liftoff-annotated gene using BEDTools intersect (version 2.31.0) [59]. Candidates were then filtered through three successive confidence criteria: (i) multi-exon structure to exclude potential single-exon artifacts; (ii) concordant prediction by both Augustus and GeneMark-ET (≥50% reciprocal overlap), ensuring independent support from two gene prediction algorithms; and (iii) evidence of active transcription, defined as overlap with at least one RNA-seq-derived splice junction (intron hint) from the BRAKER3 hints file, with a minimum of 10 total supporting RNA-seq reads across all overlapping splice junctions. To characterize the resulting candidates, protein sequences were extracted from the BRAKER3 output and queried against the NCBI nonredundant protein database using BLASTP (version 2.17.0) [43] to identify homology to known genes.

### Short-read RNA-seq and ChIP-seq data analysis

fastp (version 0.24.0) [60] was used for adapter trimming, deduplication, and overrepresented sequence analysis of the ChIP-seq and RNA-seq data. BWA (version 0.7.18-r1243-dirty) [25] was used to align the ChIP-seq reads to GRCh38 [11], to T2T-CHM13v2.0 [12], and to the post-curation diploid assemblies. HISAT2 (version 2.2.1) [61] was used to align the RNA-seq reads to GRCh38, T2T-CHM13v2.0, and to the post-curation diploid assemblies. Picard (version 3.1.1) was used to mark optical duplicates. GATK (version 4.5.0.0) [62] CollectAlignmentSummaryMetrics was used to determine the number of mapped reads (PF_READS_ALIGNED) and the mismatch rate (PF_MISMATCH_RATE).

### Short-read whole genome sequencing data analysis

BWA (version 0.7.18-r1243-dirty) [25] was used to align the Illumina read pairs separately to each post-curation haplotype. For the AS-based phasing approach, the summed alignment scores (AS tags) of R1 and R2 were computed for each haplotype, and read pairs were assigned to the haplotype with the higher total AS using a custom Python script. Read pairs were categorized into five mutually exclusive groups: “not properly mapped” if not properly mapped as a pair to either haplotype (as determined by the aligner’s proper-pair flag), “sex chromosome” if properly mapped to chrX or chrY in either haplotype, “hap1” or “hap2” if the summed AS was higher for the respective haplotype, and “homozygous” if the summed AS was equal for both haplotypes. Read pairs that were properly mapped to only one haplotype were assigned to that haplotype.

For the MAPQ-based phasing approach, read pairs were aligned to the combined diploid assembly and filtered using a mapping quality threshold (MAPQ ≥ 10) to retain only read pairs that could be confidently placed on a single haplotype. For those read pairs that could be phased by the AS-based approach, the distribution of alignment scores was plotted as a histogram. Specifically, we created a concordant distribution including all summed AS values for haplotype-concordant alignments (e.g. hap1-assigned read pairs aligned to hap1) and a discordant distribution including all summed AS values for haplotype-discordant alignments (e.g. hap1-assigned read pairs aligned to hap2). For each distribution, we fit the best exponential distribution using the statistical functions in SciPy (version 1.10.1) [63] in Python 3.11.3. We also used a two-sample Kolmogorov–Smirnov test [64] to assess whether the two distributions differed significantly.

## Results

### Long-read and chromatin conformation capture sequencing produced chromosome-level diploid genome assemblies

We first generated ONT-UL, PacBio HiFi, and Hi-C sequencing libraries from BJ and IMR-90 fibroblast cells (Supplementary Fig. S1A and Table 1). NanoPlot [22] quality control analysis indicated that these data were of high quality (Table 1 and Supplementary Fig. S1B), and *k*-mer spectra generated with GenomeScope 2.0 [21] verified the expected sequencing coverages and heterozygosity levels (Supplementary Fig. S1C). The ONT and HiFi data showed a large tail of UL reads beneficial for assembling repetitive regions (Supplementary Fig. S1D), and the Hi-C data showed a healthy distribution of long-range contacts (Supplementary Fig. S1E).

**Table 1.** Total coverage, read length N50, and median quality scores for all sequencing data

Sample	Data	Total coverage	Read length N50	Median quality score
BJ	ONT-UL	25.36× (8× ≥100kb)	66 433	20.7
BJ	HiFi	44.02×	15 421	33.2
BJ	Hi-C	25.69×	146	32.5
IMR-90	ONT-UL	21.22× (14× ≥100kb)	143 098	20.9
IMR-90	HiFi	32.32×	16 449	33.8
IMR-90	Hi-C	26.35×	146	32.7

These values were calculated using NanoPlot. Total coverage was based on the T2T-CHM13v2.0 genome size of 3 117 292 070 bp. The values for the Hi-C data were calculated after trimming five bases from the 5′ end of both read 1 and read 2 as recommended by the Arima mapping pipeline.

We produced *de novo* diploid genome assemblies using Verkko 2.2.1 [24]. Pre-curation assembly graph visualization and quality control suggested that the diploid assemblies were approaching the haploid T2T-CHM13v2.0 reference genome in quality (Supplementary Figs S2 and S3, and Supplementary Table S1). To improve the assemblies further, we manually curated both using Hi-C maps to correct misjoins and consolidate segments from the same chromosomes into single scaffolds (see the “Materials and methods” section). This refinement process substantially improved the structural quality of the assembly, ensuring that each chromosome was accurately reconstructed as a continuous and well-defined scaffold.

Following manual curation, the BJ and IMR-90 diploid assemblies spanned 5.9 and 6.0 Gbp, respectively. Both assemblies consisted of 46 scaffolds representing the 44 autosomes and two sex chromosomes (XY for BJ and XX for IMR-90). The mitochondrial genomes are not included in the chromosomal assemblies but are publicly available (see the “Data availability” section). The number of gaps were 53, 39, 41, and 29, which corresponded to <0.085%, 0.052%, 0.053%, and 0.056% of the genome length, respectively.

**Figure 1.**
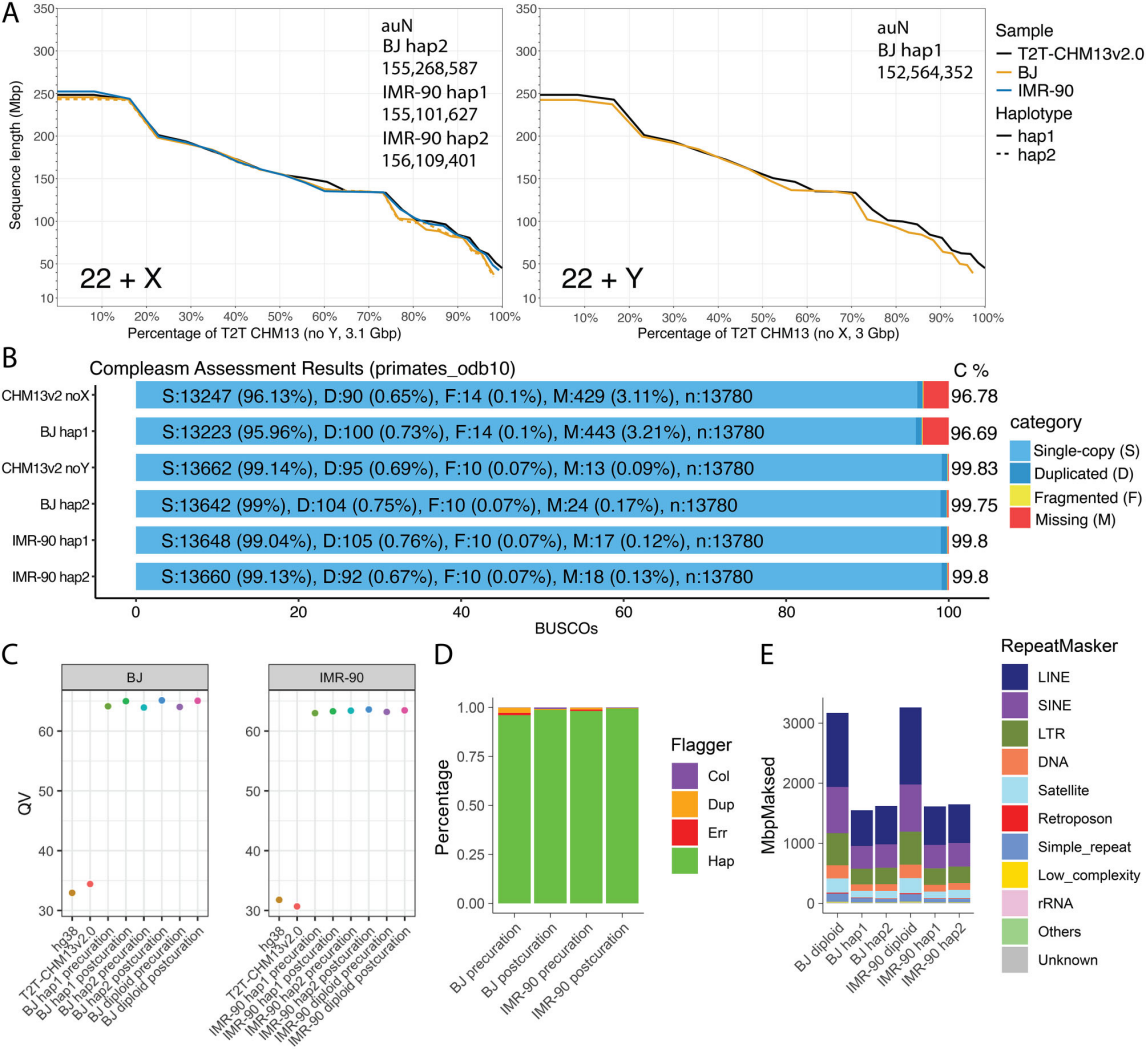
Post-curation assembly QC. (**A**) NGx charts showing the contiguity of the post-curation assembly haplotypes containing (left) chrX and (right) chrY. The NG50 value (at *x* = 50%) is the maximum sequence length such that all sequences of that length or longer together cover at least 50% of the reference genome. In black is the NGx plot for T2T-CHM13v2.0 without (left) chrY or (right) chrX. Scaffold auN values are shown in the upper right. (**B**) Compleasm results showing the estimated number of single-copy (S), duplicated (D), fragmented (F), and missing (M) near-universal single-copy ortholog gene sequences in the post-curation haplotypes. Results for T2T-CHM13v2.0 without chrX or without chrY are shown for comparison. (**C**) Merqury estimated QV of the pre- and post-curation haplotypes using hybrid databases constructed from HiFi and publicly available Illumina data. (**D**) HMM-Flagger results showing the percentage of each pre- and post-curation haplotype that is categorized as correctly assembled (hap), in error (err), falsely duplicated (dup), or collapsed (col). (**E**) RepeatMasker results showing the number of bases in the post-curation haplotypes that are categorized into each repeat family.

To quantify the contiguity of the assemblies (see the “Materials and methods” section), we calculated the auN values which represent the area under the contiguity (Nx) curve. Each haplotype-specific assembly for BJ and IMR-90 contains only a single sex chromosome, while T2T-CHM13v2.0 contains both sex chromosomes. Thus, for a fair comparison we constructed two versions of the reference: one excluding chrX (for comparison with BJ hap1) and one excluding chrY (for comparison with the other three haplotypes). The scaffold auN values of the assemblies ranged from 152.6 to 156.1 Mbp, approaching the T2T-CHM13v2.0 auN values of 154.6 Mbp (excluding chrX) and 156.5 Mbp (excluding chrY) (Table 2 and Fig. 1A). We also compared the absolute chromosome lengths of each haplotype assembly against GRCh38 and T2T-CHM13v2.0 (Supplementary Table S2). Nonacrocentric autosome lengths showed a median deviation of <1% from T2T-CHM13v2.0, with the largest differences on acrocentric chromosomes and chr9, reflecting incomplete resolution of satellite-rich pericentromeric regions. Using compleasm [29] to evaluate conserved single-copy orthologous genes, we similarly compared each haplotype to the matching single-sex-chromosome reference. BJ hap1 achieved 96.69% completeness compared to 96.78% for T2T-CHM13v2.0 excluding chrX, while BJ hap2, IMR-90 hap1, and IMR-90 hap2 achieved 99.75%, 99.80%, and 99.80% completeness compared to 99.83% for T2T-CHM13v2.0 excluding chrY (Fig. 1B).

**Table 2.** Assembly statistics for the post-curation assemblies

Summary	BJ hap1	BJ hap2	IMR-90 hap1	IMR-90 hap2
Assembled bases (bp)	2 882 767 297	2 997 324 958	2 993 756 485	3 032 665 978
Gap bases (bp)	2 461 249	1 573 056	1 577 995	1 693 070
Number of gaps	53	39	41	29
T2T contigs	2	4	3	4
T2T scaffolds	15	14	17	19
Contig NG50 (bp)	98 312 316	80 488 942	88 647 972	105 503 013
Scaffold NG50 (bp)	147 174 222	154 922 760	155 020 692	154 123 613
GC content	40.87%	40.84%	40.85%	40.81%
Telomeres	38	37	40	42
Merqury QV (*k* = 31)	64.99	65.13	63.30	63.61
T2T-CHM13v2.0 Liftoff genes	60 452	62 083	62 219	62 421
T2T-CHM13v2.0 Liftoff protein coding genes	19 195	19 904	19 902	19 827
Percent repeats	53.76%	54.08%	53.89%	54.33%
Repeat bases (bp)	1 549 698 341	1 620 816 858	1 613 390 604	1 647 684 317
LINE	20.62%	21.30%	21.38%	21.21%
SINE	13.15%	13.03%	13.07%	12.95%
LTR	9.05%	9.12%	9.15%	9.08%
Satellite	3.89%	4.26%	3.80%	4.67%
DNA	3.70%	3.72%	3.73%	3.70%
Simple repeat	2.63%	1.96%	2.04%	2.00%
Low complexity	0.20%	0.20%	0.20%	0.20%
Retroposon	0.25%	0.24%	0.25%	0.24%
rRNA	0.01%	0.01%	0.01%	0.01%

BJ hap1 includes chrY and BJ hap2 includes chrX. Each haplotype of IMR-90 includes one copy of chrX. The assembled bases, gap bases, number of gaps, contig and scaffold NG50s, and GC content were calculated using gfastats. The QV scores were calculated using Merqury with HiFi and publicly available Illumina data. The T2T-CHM13v2.0 gene annotation was lifted over to each post-curation assembly haplotype using Liftoff. The repeat content was calculated using RepeatMasker (see the “Materials and methods” section).

We next assessed assembly correctness using Merqury [31] with hybrid *k*-mer databases (*k* = 31) constructed from HiFi and publicly available Illumina whole genome sequencing data (Fig. 1C). The BJ and IMR-90 assemblies achieved diploid QV scores of 65.06 and 63.46, corresponding to completeness values of 99.68% and 99.72%, respectively. As a point of comparison, we also evaluated the unmatched reference genomes GRCh38 and T2T-CHM13v2.0 against the same hybrid *k*-mer databases using Merqury, which yielded QV scores ranging from 30.68 to 34.43. These lower scores do not reflect the quality of the reference genomes themselves but rather confirm that the BJ and IMR-90 assemblies faithfully capture the sample-specific variants and genomic structures of their respective cell lines.

We then used Flagger [13] to determine the percentage of each assembled haplotype that is categorized as correctly assembled (hap), in error (err), falsely duplicated (dup), or collapsed (col) (Fig. 1D). The final BJ and IMR-90 assemblies were predicted to be over 98% and 99% correctly assembled, respectively. Final inspection of the Hi-C contact map showed characteristic diamond shapes for each of the chromosomes reflecting spatially segregated haplotypes and did not reveal any off-diagonal contacts, confirming the correct order and orientation of all scaffolds (Supplementary Fig. S4).

We next used RepeatMasker to locate and quantify the repeat families within each of the assembled haplotypes (Fig. 1E). The number of bases in each category closely matched the corresponding numbers in T2T-CHM13v2.0, with LINEs comprising around 21% of each haplotype, and SINEs comprising around 13%. LTRs comprised the next most prevalent repeat family, with around 9% in each haplotype.

To characterize the repetitive content across each haplotype assembly, we generated alignment-free low-complexity maps using AlcoR [37]. AlcoR uses bidirectional data compression with finite-context models to identify LCRs without requiring a reference genome or repeat library. By varying the memory parameter of the context models, we distinguished three classes of LCRs based on the spatial distance between repeated patterns: local LCRs (repeats within 5 kb, e.g. tandem repeats and microsatellites), medium-range LCRs (repeats within 50 kb, e.g. tandem gene arrays and larger satellite blocks), and distant LCRs (repeats found anywhere across the chromosome, e.g. segmental duplications and interspersed elements). Each class was assigned a distinct color (red, green, and blue, respectively) and visualized as haplotype-resolved ideogram-style maps (Supplementary Fig. S5). As expected, centromeric and pericentromeric regions exhibited the highest density of LCRs across all three distance classes, consistent with the known enrichment of satellite DNA arrays in these regions, while distant LCRs were distributed across the chromosome arms, corresponding to known segmental duplications and interspersed repeat families.

### Computational karyograms confirm the structural integrity of the assemblies

**Figure 2.**
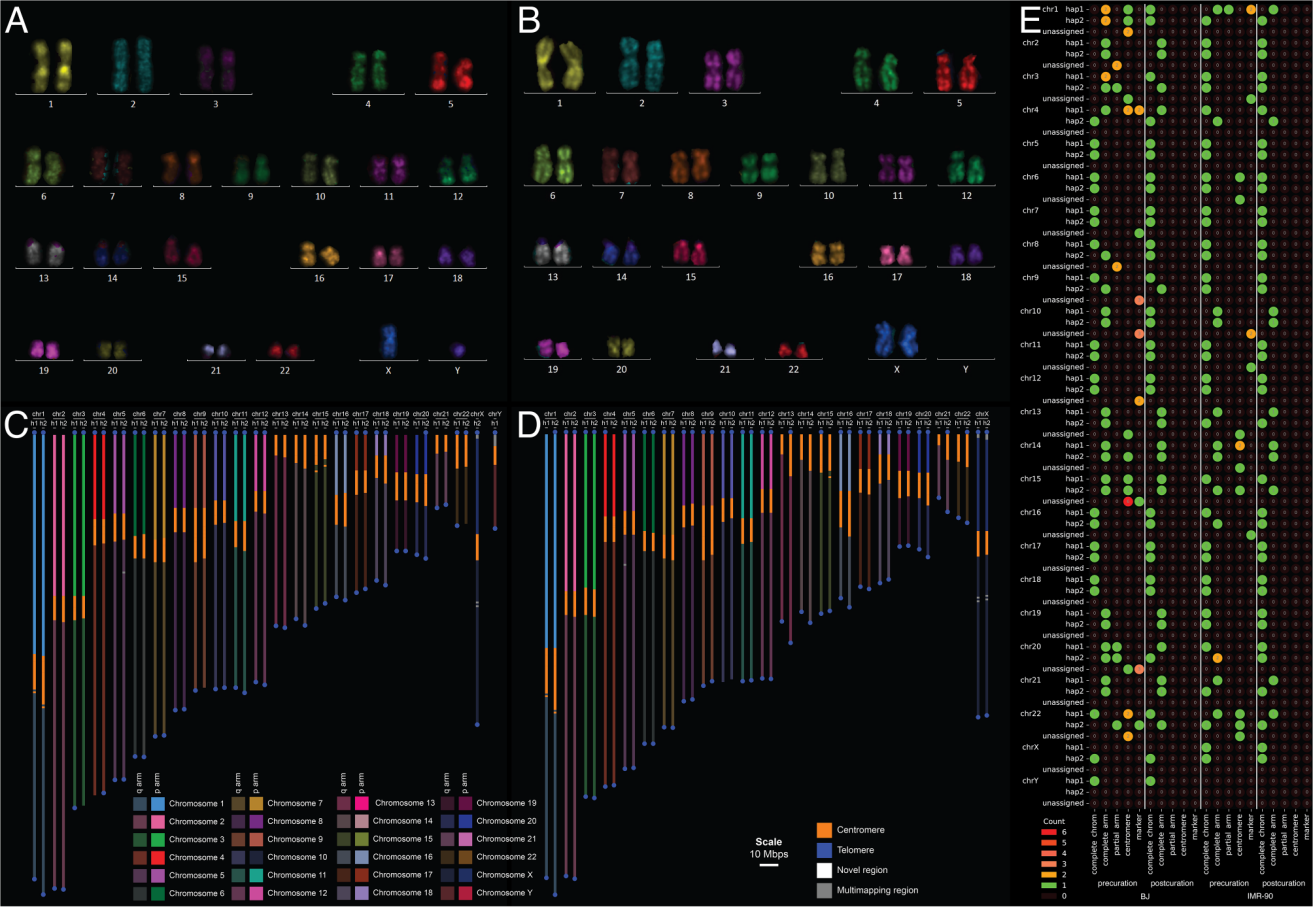
Structural completeness of the post-curation assemblies. (**A, B**) Karyograms of the BJ and IMR-90 cell lines, respectively, acquired using the MetaSystems Metafer Slide Scanning Platform and Ikaros module. (**C, D**) KaryoScope computational karyograms of the BJ and IMR-90 post-curation assemblies. (**E**) Status of completeness for the pre- and post-curation assemblies. Each contig/scaffold of the assembly is categorized as complete chromosome, complete arm, partial arm, centromere, or marker.

To evaluate the structural completeness of the assemblies, we compared them to traditional karyotypes. M-FISH karyotyping of BJ and IMR-90 cells confirmed that both lines were diploid and 46,XY and 46,XX, respectively (Fig. 2A and B). To facilitate the comparison of traditional karyotypes and assembled genomes, we developed KaryoScope. KaryoScope annotates DNA sequences in an alignment-free manner by querying them against a database that associates each short DNA segment (*k*-mer) with a corresponding genomic feature. KaryoScope is inspired by traditional cytogenetics and FISH, but unlike optical methods, there is no practical limit to the number of genomic features that can be queried.

The KaryoScope *k*-mer database associates all the 31-mers in T2T-CHM13v2.0 to one of the following features: telomere if the 31-mer occurs in the telomere region as defined by seqtk, centromere if it occurs in the centromere region as defined by the CenSat annotation [65], or chromosome arm (e.g. chr1p, chr1q) if it maps to one of the 48 chromosome-specific arms. KaryoScope can be used to query any genome assembly against this database to assign contigs to chromosomes, orient the contigs, and order the contigs. Each feature is assigned a color, and KaryoScope plots every sequence (e.g. contig or scaffold) in the assembly as a colored vertical rectangle to identify the positions of each feature (see the “Materials and methods” section).

As a visual quality control, we applied KaryoScope to the pre- and post-curation assemblies (Supplementary Fig. S6 and Fig. 2C and D). Although the pre-curation assemblies were broken at several centromeres, the post-curation assemblies were almost entirely telomere-to-telomere. Some chromosomes were missing telomeres in the final assemblies, particularly at the acrocentric p-arms, but were otherwise complete. Overall, the genome structure observed in the genome assemblies closely mirrored what we saw in the traditional karyotypes, with the KaryoScope computational karyograms confirming that the final assemblies consisted of one scaffold per pseudomolecule.

To quantify structural completeness, we categorized every contig or scaffold of the pre- and post-curation assemblies into one of five categories. “Complete chromosome” refers to fully telomere-to-telomere sequences. “Complete arm” refers to sequences with at least one complete arm flanked by centromeric sequence on one side and telomeric sequence on the other. The categories “partial arm,” “centromere,” and “marker chromosome” refer to (i) arm-specific sequences flanked on only one side with telomeric or centromeric sequence, (ii) entirely centromeric sequences, or (iii) arm-specific sequences without any flanking telomeric or centromeric sequence, respectively. For the post-curation assemblies, all scaffolds were either classified as either “complete chromosome” when fully telomere-to-telomere, or “complete arm” when missing only a single telomere (Fig. 2E and Supplementary Table S3).

### *De novo* assemblies resolve complex centromere and telomere regions

**Figure 3.**
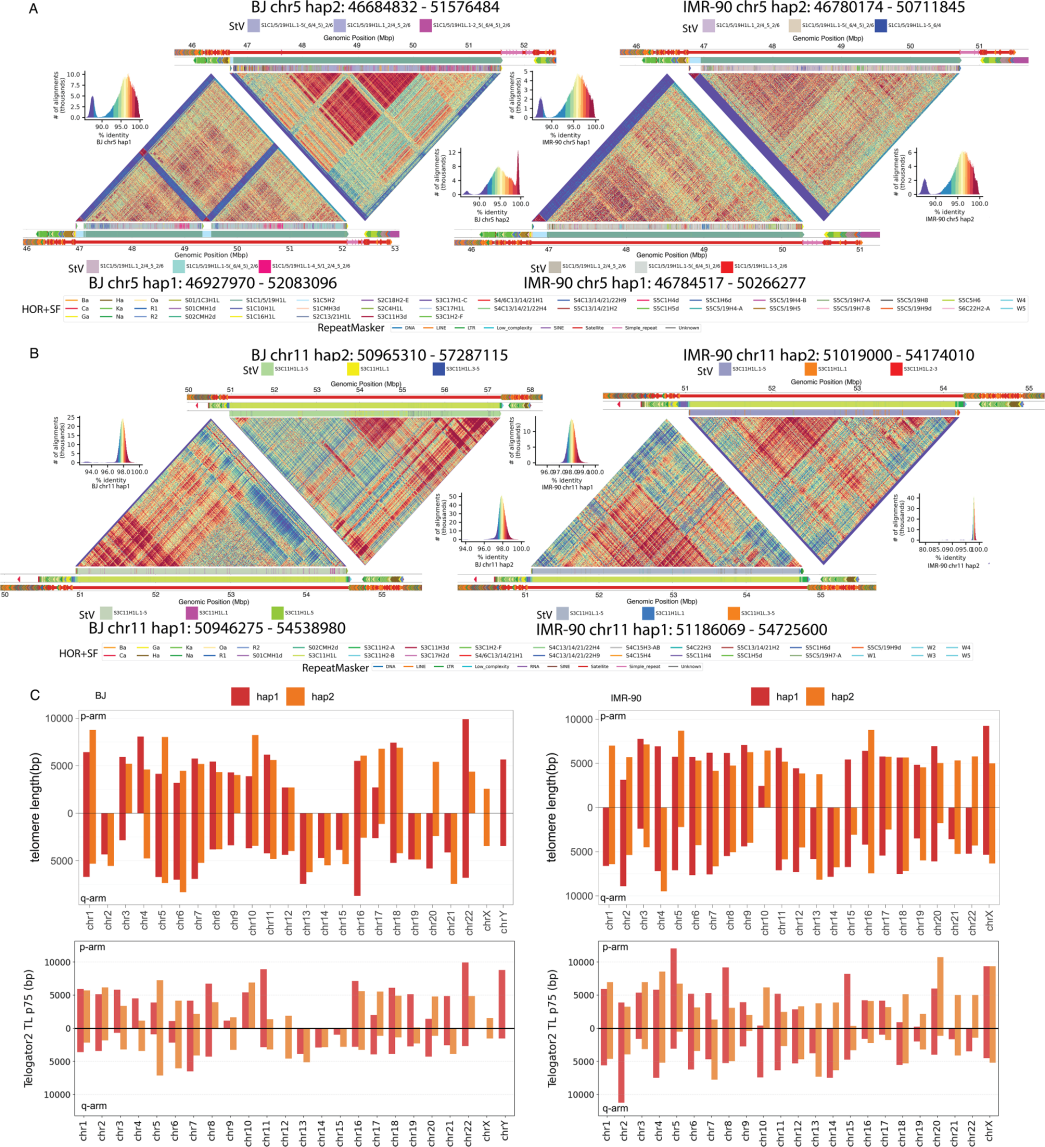
Centromeres and telomeres in the post-curation assemblies. (**A, B**) StainedGlass plots of BJ and IMR-90 α-satellite HORs within the chr5 and chr11 centromeres. The colors in each heatmap are based on sequence alignment percent identity (shown in the histograms), with red being the highest level of sequence similarity. The innermost annotation track consists of the live HORs only. Each color represents a different StV variant of the HOR monomers. The top three most abundant variants within each centromere are labeled. The central annotation track is an extended region including 1Mbp upstream and downstream of the live HORs. Each color represents a different HOR or other repeat superfamily (HOR + SF). The outermost annotation track consists of the repeat classes from RepeatMasker. (**C**) The upper panels show bar plots of arm-specific telomere lengths for BJ (left) and IMR-90 (right) in the final version of the assemblies. The lower panels show corresponding 75th percentile telomere lengths for BJ (left) and IMR-90 (right) estimated by telogator2 directly from long-read data.

Due to their highly repetitive nature, centromeres are difficult to assemble and have routinely been omitted from reference genomes, though this has changed with the efforts of the T2T and HPRC consortia [66, 67]. To validate the quality of our centromere assemblies, we evaluated each assembly with Flagger, which showed that 21 and 35 of the 48 centromeric HOR arrays were correctly assembled in BJ and IMR-90, respectively. For additional validation, we analyzed centromeric sequence homology using StainedGlass [39] for both haplotypes of BJ and IMR-90 (chromosomes 5 and 11 shown in Fig. 3A and B). From the StainedGlass plots, we found that the structures of the functionally active HOR arrays were conserved between the two cell lines, and that the level of centromere completeness in the final assemblies was comparable to the centromere structures in T2T-CHM13v2.0 [66].

The immediate flanking satellite sequence upstream and downstream of the active HOR arrays was conserved both within each chromosome and relative to T2T-CHM13v2.0. These results indicate that some regions within human centromeres are highly conserved. This is not surprising as disruptions within the functional centromere might impact kinetochore formation with detrimental consequences for cell division. Within the active HOR arrays, we annotated the different alpha satellite variants and found that the two haplotypes in BJ showed more differences in HOR unit arrangement than those in IMR-90, suggesting different haplogroups of origin for the two BJ haplotypes. We provided further annotation and characterization of centromere organization using the Centeny Maps pipeline [40], which shows consistent representation of CENP-B box patterns between the two haplotypes for both BJ and IMR-90 (Supplementary Fig. S8). The tracks from the GCP pipeline [40] are also provided as additional annotation layers to further explore centromere and whole-genome architecture of these assemblies.

Like centromeres, telomeres and subtelomeres have long been excluded from human reference genomes. The assemblies resolved 75 and 82 telomeres in BJ and IMR-90, respectively, out of 92 chromosome arms (Fig. 3C). Many of the missing telomeres were on the acrocentric p arms, with 8 and 6 of the 10 acrocentric telomeres missing in BJ and IMR-90, respectively. To better assess the assembly quality in the telomeric regions, we included telomere length estimates from telogator2 [41] for comparison.

We found no haplotype-specific differences in assembly-based telomere lengths within either cell line. IMR-90 telomeres were slightly longer than those in BJ, possibly suggesting that the BJ cells spent more time in culture prior to our handling (Supplementary Fig. S7A). Assembly-based telomere lengths for both cell lines fell at the lower end of the distribution observed across the HPRC cohort (Supplementary Fig. S7B and Supplementary Table S4), likely reflecting the extensive culture history of these cell lines compared to the typically low-passage HPRC samples. This trend is further illustrated within the HPRC cohort itself, where samples showed shorter assembly-based telomere lengths in later years of the project. To assess the accuracy of assembly-based telomere length estimates, we used telogator2 to estimate telomere lengths of 49 HPRC samples directly from long-read sequencing data. Assembly-based telomere lengths correlated well with the read-based telogator2 estimates (Supplementary Fig. S7C and Supplementary Table S5), suggesting that telomere lengths can be reliably estimated directly from assemblies.

To assess whether any viral sequences were integrated into the assemblies, we first screened all four haplotype assemblies using the NCBI FCS tool for adaptor, vector, and nonhuman sequence contamination. No contaminant sequences were identified. We then performed BLASTn screening of 12 viral genomes known to integrate into telomeric or subtelomeric regions [68] (B19V, HSV-1, VZV, EBV, HCMV, HHV-6B, HHV-7, JCPyV, MCPyV, HPyV6, HPyV7, and HPV) against all four haplotype assemblies (Supplementary Table S6). No evidence of viral integration was found. Ten viruses produced no significant hits at stringent thresholds (≥90% identity, ≥500 bp). HHV-6B and HHV-7 produced hits exclusively within annotated telomeric regions, corresponding to (TTAGGG)n repeat homology in the direct repeat (DR) regions of these Roseolovirus genomes rather than genuine viral integration.

### Structural comparisons of the assemblies reveal large scale variations within and between cell lines

**Figure 4.**
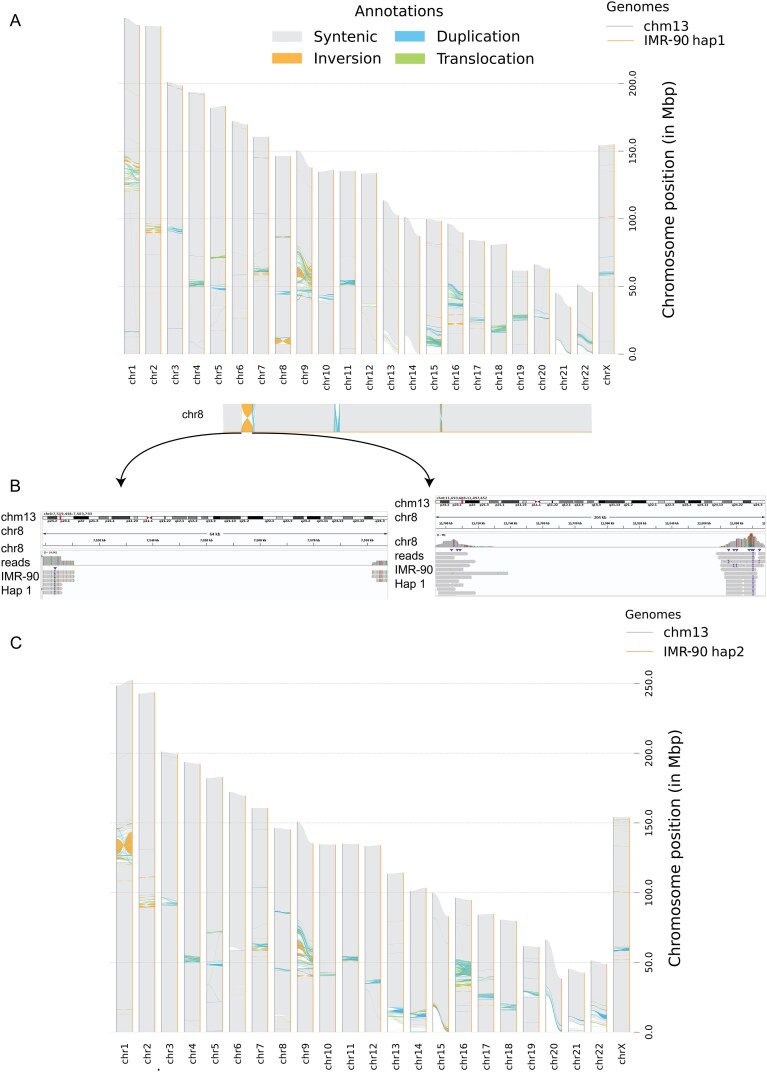
Example haplotype-specific inversion on chromosome 8 in IMR-90. (**A**) SyRI synteny plot of IMR-90 haplotype1 against T2T-CHM13v2.0. Inversions are in orange color. At the bottom is the zoomed-in view of the example inversion. (**B**) IGV screenshots of IMR-90 reads aligned to T2T-CHM13v2.0 chromosome 8 at both breakpoints of the inversion. (**C**) SyRI synteny plot of IMR-90 haplotype2 against T2T-CHM13v2.0 showing no inversion in chromosome 8.

Multisample synteny analysis of the 22 autosomes across the assemblies and T2T-CHM13v2.0 [44] revealed a high degree of collinearity of the two *de novo* diploid assemblies (Supplementary Fig. S9A). Both BJ and IMR-90 contained a large haplotype-specific inversion on chromosome 8 (hap1) when compared to T2T-CHM13v2.0. The inversion was present in only one haplotype, as shown for IMR-90 by synteny analysis of each haplotype against the reference (Fig. 4A and C) and by inspection of read alignments at both breakpoints (Fig. 4B). The inversion appears to correspond to a well-known polymorphic inversion at 8p23, which has an allele frequency of ~50% [69, 70] and has been associated with miscarriages when carried by one parent [71]. To check for large-scale haplotype-specific SVs within each cell line, we aligned both haplotypes to each other and visualized the alignments using SVbyEye [45] (Supplementary Fig. S9B and C). Although the inversions on chromosome 8 are clearly visible, no other obvious large-scale haplotype-specific variation could be observed.

**Figure 5.**
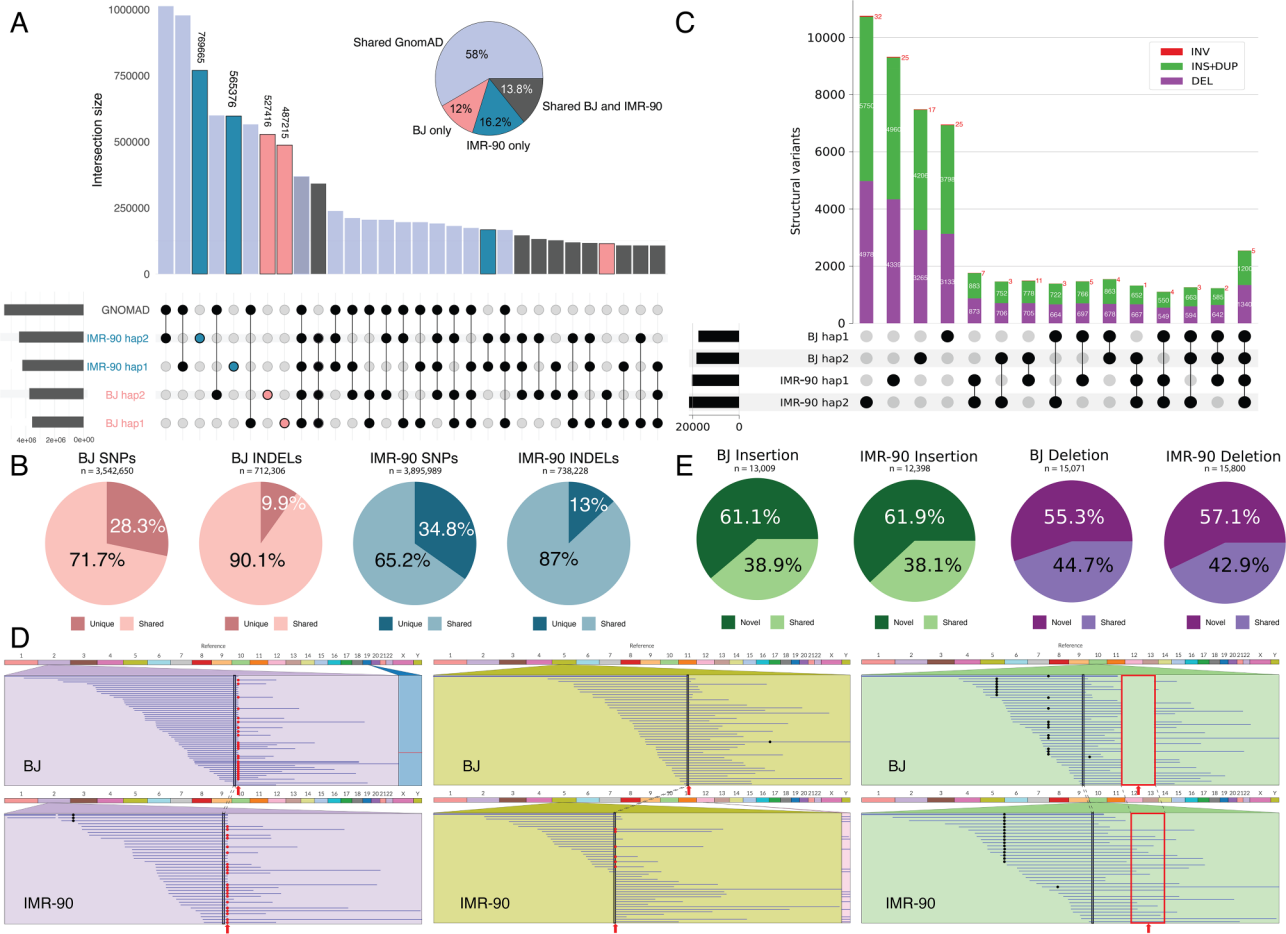
Variation in the post-curation assemblies. (**A**) UpSet plot showing the number of SNVs and indels across the four post-curation haplotypes and gnomAD. The pie chart shows the proportion of BJ and IMR-90 variants that are shared with gnomAD. (**B**) The percentages of BJ and IMR-90 SNVs and indels that are cell-line-specific or are shared within gnomAD. (**C**) UpSet plot showing the number of SVs by type across the four post-curation haplotypes. (**D**) Three example SVs: ribbon plot of a 4636 bp insertion on chromosome 2 shared by all four haplotypes; ribbon plot of a 4459 bp insertion on chromosome 5 present only in IMR-90; ribbon plot of a 4342 bp deletion on chromosome 10 present only in BJ. Red arrows indicate the breakpoint coordinates in each panel. Insertions appear as red dots in the aligned reads of carrier haplotypes, while the deletion is visible as a gap in read coverage in BJ but not in IMR-90. (**E**) The percentages of BJ and IMR-90 SV insertions and deletions that are cell-line-specific or are shared within the pangenome.

To systematically call variants directly from the assemblies, we used SyRI [46] to call SNVs, indels, and SVs within each of the four assembled haplotypes compared to T2T-CHM13v2.0 (Fig. 5). After merging small variants (SNVs and indels) from the four haplotypes, we found *n* = 7 786 003 SNVs and *n* = 1 486 112 indels. Amongst all small variants found in BJ (*n* = 5 611 994) and IMR-90 (*n* = 6 842 223) (Fig. 5A), *n* = 4 003 178 (71.3%) and *n* = 5 111 723 (74.7%) were heterozygous, respectively. A total of *n* = 3 182 102 small variants (56.7% of BJ and 46.5% of IMR-90) were shared between cell lines. Conversely, *n* = 2 429 892 (43.3%) of small variants found in BJ were specific to BJ whereas *n* = 3 660 121 (53.5%) of those found in IMR-90 were specific to IMR-90. To confirm whether the BJ and IMR-90 SNVs and indels were novel or present in the general population, we compared them to gnomAD [47] (Fig. 5B). We found that 71.7% of SNVs in BJ, 90.1% of indels in BJ, 65.2% of SNVs in IMR-90, and 87% of indels in IMR-90 were previously noted in the gnomAD database.

We next sought to characterize large variants (SVs) found in the assemblies. After merging the four sets of SVs using Jasmine [48], there were *n* = 51 105 SVs, including *n* = 27 128 insertions, *n* = 23 830 deletions, and *n* = 147 inversions. Amongst all BJ (*n* = 29 258) and IMR-90 (*n* = 35 116) SVs (Fig. 5C), *n* = 22 679 (77.5%) and *n* = 28 385 (80.8%) were heterozygous (and therefore haplotype-specific), respectively. A total of *n* = 13 269 SVs (45.4% of BJ and 37.8% of IMR-90) were shared between cell lines. Conversely, *n* = 15 989 (54.6%) of SVs found in BJ were specific to BJ whereas *n* = 21 847 (62.2%) of SVs found in IMR-90 were specific to IMR-90, suggesting that most SVs were cell-line-specific. These results demonstrate that most SVs detected were heterozygous and cell-line-specific.

Our analysis of these variants was based on direct inference from the assembled haplotypes. To validate the SVs using traditional alignment-based methods, we investigated three specific examples by plotting read alignments across the SV breakpoints (Fig. 5D). We selected a 4636 bp insertion on chromosome 2 shared by all four haplotypes, a 4459 bp insertion on chromosome 5 present in both haplotypes of IMR-90, and a 4342 bp deletion on chromosome 10 present in both haplotypes of BJ. To characterize the two insertions, we performed BLASTn searches against the NCBI nucleotide collection (nr/nt). The 4636 bp insertion on chromosome 2 is 99.98% identical to BAC RP11-514N8 (AC069162.8) from the chromosome 2q13–2q14.1 ancestral fusion site, a region enriched in segmental duplications and subtelomeric-derived sequence. The 4459 bp insertion on chromosome 5 is 100% identical to a previously characterized nonreference unique insertion at chr5:75 128 425 (MH534391.1) [72], representing human sequence absent from T2T-CHM13v2.0. Both insertions are thus known human SVs rather than foreign sequences.

We also performed independent alignment-based SV calling using Sniffles2 [52] with combined HiFi and ONT reads aligned to T2T-CHM13v2.0. Sniffles2 detected 31 588 (BJ) and 38 379 (IMR-90) SVs at least 50 bp in length, while assembly-based calling with SyRI identified comparable totals of 29 258 and 35 116 SVs, respectively, with similar per-type distributions of deletions and insertions (Supplementary Table S7). The assembly-based approach additionally resolved SV types that are underrepresented in alignment-based calling; for example, SyRI identified 918 (BJ) and 1178 (IMR-90) duplications compared to 27 and 35 by Sniffles2, and 83 and 101 inversions compared to 30 and 40. Notably, when the same reads were aligned to each sample’s matched diploid assembly rather than T2T-CHM13v2.0, the number of SVs detected against the matched reference was reduced by 92.7% for BJ (from 31 588 to 2297) and 97.1% for IMR-90 (from 38 379 to 1097), demonstrating that the assemblies capture nearly all structural variation present in each cell line.

Having established the SV landscape for BJ and IMR-90, we compared our large variant calls against SVs from the Year 1 HPRC pangenome (*n* = 44). After merging large insertions and deletions, we found that 41% of SVs in BJ and IMR-90 were also present in the HPRC call set (Fig. 5E), indicating that many of the BJ and IMR-90 variants are shared with the broader population. The lower proportion of shared SVs compared to SNVs and indels found in gnomAD likely reflects the smaller size of the Year 1 HPRC cohort relative to the 76 156 individuals in gnomAD.

We next categorized HPRC samples by the number of SV insertions and deletions shared with BJ and IMR-90 (Supplementary Fig. S10A). BJ shared the most variants with admixed American (AMR) samples, while IMR-90 shared the most with African (AFR) samples. This prompted us to perform a genome-wide ancestry analysis of the BJ and IMR-90 assemblies (Supplementary Fig. S10B and C). Although most of both genomes were assigned as European, IMR-90 contained more genomic regions categorized as AFR than BJ.

Intriguingly, HPRC samples showed on average 7509 (26.58%) unique SVs not detected in either BJ or IMR-90 (Supplementary Fig. S10A), contrasting with the 14 805 (58%) and 18 357 (59%) SVs uniquely observed in BJ and IMR-90, respectively, and absent from all HPRC samples. This higher variant burden in the cell lines potentially reflects variation acquired *in vitro*, motivating cell-line assembly and underlining the importance of this work.

### *De novo* gene prediction identifies gene duplications missed by reference-based annotation

To comprehensively annotate each haplotype, we combined reference-based annotation mapping using Liftoff [35] with *de novo* gene prediction using BRAKER3 [58] (see the “Materials and methods” section). BRAKER3-predicted genes that did not overlap any Liftoff annotation and passed filters for multi-exon structure, concordant prediction by both Augustus and GeneMark, and RNA-seq support were identified as putative novel gene candidates for further characterization. We identified 41, 18, 7, and 12 candidates in BJ hap1, BJ hap2, IMR-90 hap1, and IMR-90 hap2, respectively (Supplementary Table S8). BLAST analysis revealed that 73 (94%) of these 78 candidates represent known human genes or additional copies of known gene families that Liftoff failed to place, rather than truly novel genes. Two had low-identity BLAST hits to nonprimate organisms, and three had no hits at all. These five all localize to subtelomeric regions or regions rich in segmental duplications, where rapid structural evolution may give rise to lineage-specific or highly divergent gene copies that lack representation in current databases.

A cluster of additional gene copies on chromosome 7q22.1 was detected across all four assemblies, comprising extra copies of *RASA4, RASA4B, UPK3BL1*, and *SPDYE2B*. This locus contains a known copy-number variable segmental duplication with 1–7 *RASA4* copies per allele in the population, which has been associated with type 2 diabetes risk and chronotype [73]. Copy number varied across haplotypes (Table 3), with BJ hap1 showing the most pronounced expansion (12 total *RASA4*/*RASA4B* copies compared to 3 placed by Liftoff), and all additional copies were confirmed to be transcriptionally active by RNA-seq.

**Table 3.** Expanded gene families at 7q22.1 and 16p11.2 loci in BJ and IMR-90

Locus	Gene	BJ hap1	BJ hap2	IMR-90 hap1	IMR-90 hap2
7q22.1	*RASA4*	10	3	3	3
7q22.1	*RASA4B*	2	1	1	1
7q22.1	*UPK3BL1*	2	1	2	3
7q22.1	*SPDYE2B*	5	1	1	2
16p11.2	*SEZ6L2*	3	3	1	1
16p11.2	*ASPHD1*	3	3	1	1
16p11.2	*KCTD13*	3	3	1	1
16p11.2	*CDIPT*	2	2	1	1
16p11.2	*MAPK3*	2	2	1	1
16p11.2	*TBX6*	2	2	1	1

Most notably, both BJ haplotypes carried nine additional gene copies at the 16p11.2 locus spanning ~1 Mbp, including extra copies of *SEZ6L2, ASPHD1, KCTD13, CDIPT, MAPK3*, and *TBX6*. All were confirmed by BLAST to have at least 99.9% protein identity and were supported by up to 9298 RNA-seq reads per gene. Neither IMR-90 haplotype contained additional copies at this locus. The nearly identical gene content and expression levels across both BJ haplotypes are consistent with a homozygous duplication at 16p11.2. To our knowledge, this duplication has not been previously reported in the BJ cell line, which has been characterized as karyotypically normal by conventional cytogenetics (ATCC CRL-2522), a method with insufficient resolution to detect submicroscopic rearrangements of this size. The 16p11.2 region is one of the most well-characterized recurrent CNV loci in the human genome, with deletions and duplications at this locus associated with neurodevelopmental phenotypes including autism spectrum disorder and schizophrenia [74, 75].

These results demonstrate the complementary value of combining reference-based and *de novo* annotation approaches, particularly at structurally variable loci where reference-based mapping is inherently limited by the copy number present in the reference.

### Sample-matched reference genomes vastly improve read mapping and phasing of short read (epi)genome and expression datasets

To establish the benefit of using high-quality sample-matched assemblies relative to nonmatching reference genomes, we performed traditional alignment-based sequencing analysis using outside datasets that were not used to generate the assemblies. To this end, we downloaded publicly available BJ and IMR-90 whole genome sequencing data from NCBI, ChIP-seq data from ENCODE, and generated 286 million and 138 million read pairs of RNA-seq data in-house for BJ and IMR-90, respectively (see the “Data availability” section).

First, we asked whether short-read data could be phased by leveraging the haplotype-resolved structure of our diploid assemblies. We evaluated two alignment-based phasing approaches (see the “Materials and methods” section). In the first approach, read pairs were aligned to the combined diploid assembly and filtered using a mapping quality threshold (MAPQ ≥ 10) to retain only the read pairs that could be confidently placed on a single haplotype. This approach phased 19.1% and 25.6% of the 150 bp paired-end Illumina read pairs for BJ and IMR-90, respectively. However, in regions where the two haplotypes are similar but not identical, the mapping quality of both alignments will be low, even when small sequence differences between haplotypes could in principle distinguish the read’s origin.

To recover these reads, we developed a second approach based on alignment scores (AS). For each read pair, we computed the sum of R1 and R2 alignment scores for each haplotype and assigned the read pair to the haplotype with the higher total AS. Read pairs were categorized into five mutually exclusive groups: “not properly mapped” (not properly mapped as a pair to either haplotype, as determined by the aligner’s proper-pair flag), “sex chromosome” (properly mapped to chrX or chrY in either haplotype), “hap1” or “hap2” (higher summed AS for the respective haplotype), and “homozygous” (equal summed AS for both haplotypes). Read pairs that were properly mapped to only one haplotype were assigned to that haplotype. Sex chromosome and homozygous read pairs were excluded from haplotype-specific analyses.

**Figure 6.**
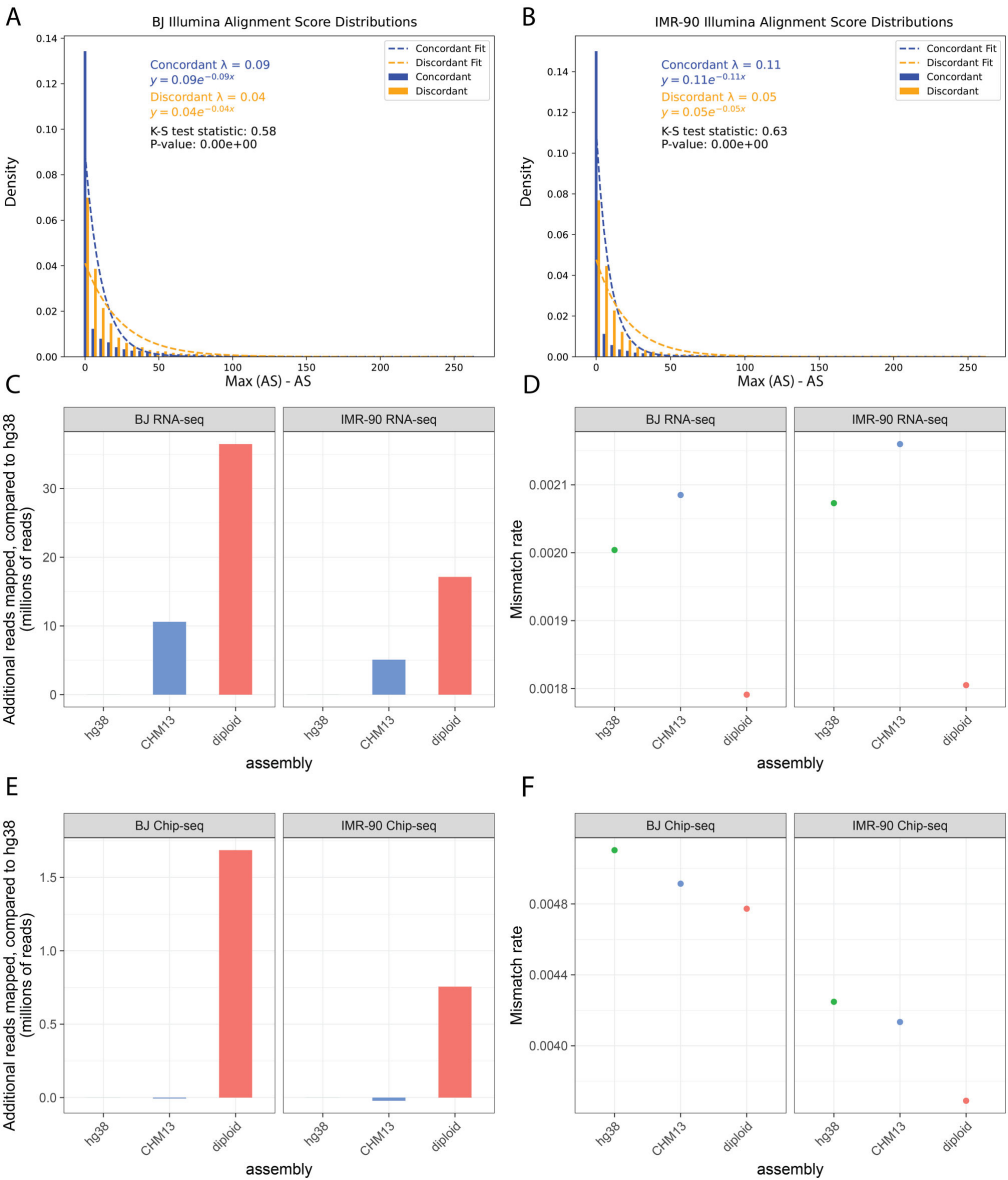
External validation of the post-curation assemblies. Exponential distributions of haplotype-concordant and haplotype-discordant alignment scores for publicly available Illumina read pairs mapped to the matched diploid assemblies, for (**A**) BJ and (**B**) IMR-90. The two distributions are significantly different (two-sample Kolmogorov–Smirnov test). Bar charts showing the number of additional mapped reads compared to GRCh38 for (**C**) RNA-seq data and (**E**) ChIP-seq data. Dot plots showing the mismatch rate of the aligned reads for (**D**) RNA-seq data and (**F**) ChIP-seq data.

To validate the AS-based phasing, we compared alignment scores for haplotype-concordant versus haplotype-discordant alignments. Here, a haplotype-concordant alignment refers to a read pair aligned to its AS-assigned haplotype (e.g. a read pair assigned to hap1 aligned against the hap1 assembly), whereas a haplotype-discordant alignment refers to the same read pair aligned to the opposite haplotype. We found that alignment score distributions were significantly different between concordant and discordant alignments (two-sample Kolmogorov–Smirnov test; Fig. 6A and B). The median concordant alignment score was higher than the median discordant alignment score for both BJ (302 versus 292) and IMR-90 (300 versus 290). In BJ, 4 million more read pairs achieved the maximum alignment score in the concordant alignment (Supplementary Fig. S11). Overall, the AS-based approach phased 23.2% and 27.5% of the Illumina read pairs for BJ and IMR-90, respectively (Supplementary Table S9), demonstrating that straightforward read alignment to a matching diploid assembly enables haplotype-level analysis of standard short-read datasets.

Next, we aligned 150 bp paired-end BJ and IMR-90 RNA-seq data to GRCh38, T2T-CHM13v2.0, and the matching *de novo* diploid assembly. After filtering for primary alignments only, we counted the number of reads mapped. Compared to GRCh38, T2T-CHM13v2.0 performed slightly better, with over 10 million and 5 million more reads mapped for BJ and IMR-90, respectively (Fig. 6C). The matched diploid assembly yielded even better results, with over 36 million and 17 million additional reads properly mapped when compared to GRCh38 for BJ and IMR-90, respectively. Among the subset of RNA-seq reads that mapped to all assemblies, we observed the lowest mismatch rates in reads mapped to the matched assembly, suggesting that sample-matched assemblies provide both improved mappability and higher-confidence alignments (Fig. 6D).

Finally, we observed a similar trend examining alignment results of publicly available 36 bp single-end ChIP-seq data. Sample-matched diploid assemblies outperformed standard reference genomes in terms of both the number of reads aligned and the mismatch rates of aligned reads (Fig. 6E and F). In conclusion, sample-matched diploid assemblies can substantially improve the quality of downstream functional analyses.

## Discussion

Our study presents the first diploid *de novo* chromosome-scale genome assemblies of the widely used human fibroblast cell lines BJ and IMR-90. To validate the structural integrity of these assemblies, we developed KaryoScope, an alignment-free tool that uses *k*-mer databases to rapidly annotate and visualize genomic features. While we used KaryoScope here as a visual quality control, its flexible framework could be applied more broadly to screen for contamination or misassembly, or to compare assemblies across cell line passages to detect acquired structural changes. Beyond structural validation, combining reference-based and *de novo* gene annotation revealed clinically relevant copy number variation missed by standard approaches, including a previously unreported homozygous duplication at 16p11.2 in BJ cells. This finding demonstrates that even cell lines characterized as karyotypically normal by conventional cytogenetics can harbor submicroscopic rearrangements with potential functional consequences, underscoring the value of complete diploid assembly for widely used model systems. Importantly, the assemblies facilitate improved read mapping and phasing, enabling new insights from the extensive sequencing data generated for these lines over the past several decades.

We selected BJ and IMR-90 fibroblasts for this study because they are genetically unperturbed, nonimmortalized cell cultures that were directly derived from primary patient materials. Both lines harbor functional cell-cycle checkpoints and undergo telomere-dependent replicative senescence making them physiologically relevant models for studying cellular aging and telomere biology.

This distinguishes these assemblies from ongoing efforts to establish phased, chromosome-scale genome assemblies from transformed or immortalized cells, which are often used to ensure ample input material. The two most common approaches are transformation of B cells to generate lymphoblastoid cell lines and viral transduction of human telomerase reverse transcriptase (hTERT), but these are not without genomic consequences. EBV-transformed cells can acquire chromosomal instability, telomere elongation, and altered gene expression, and these effects persist even with minimal post-transformation passaging. hTERT immortalization drives constitutive telomerase activity, potentially leading to substantial additions of canonical telomeric repeats at chromosome ends. The BJ and IMR-90 assemblies presented here therefore represent the first long-read assemblies of genetically unperturbed human cell lines with finite replicative potential.

A critical advance of this study is the application of the assemblies to historical sequencing data. Using straightforward alignment to the matched diploid genomes, we phased 23%–28% of publicly available short reads and recovered over 36 million additional mapped RNA-seq reads for BJ compared to GRCh38. These improvements extend to epigenomic data, with ChIP-seq alignments showing reduced mismatch rates and increased read counts when using the matched assemblies. Given that BJ and IMR-90 have been profiled extensively by ENCODE, the 4D Nucleome Project, and numerous independent laboratories, the cumulative impact of improved mapping across these datasets is substantial. Our alignment-based phasing approach is intentionally simple; future pangenome graph-alignment methods will almost certainly enable even more comprehensive reanalysis of these data.

These assemblies also have practical implications for genome editing applications. CRISPR guide RNA design tools typically evaluate on-target efficiency and off-target risk against a single haploid reference, which does not account for sample-specific variants that may create or destroy guide binding sites. In BJ and IMR-90, we identified thousands of cell-line-specific SNVs and indels, any of which could alter the seed region or PAM sequence of a candidate guide RNA. Heterozygous variants are particularly consequential, as they can lead to differential cutting efficiency between alleles, which is an effect invisible when designing guides against GRCh38 or T2T-CHM13v2.0 alone. Because the assemblies are phased, researchers can design haplotype-specific guide RNAs that selectively target one allele while leaving the other intact, enabling allele-specific knockouts or precise monoallelic editing strategies that are not feasible with a haploid reference. For example, the homozygous duplication at 16p11.2 we identified in BJ means that CRISPR experiments targeting genes such as *MAPK3* or *KCTD13* in this cell line would need to account for additional gene copies missing from the reference, potentially altering both the number of target sites and the interpretation of knockout efficiency. Similarly, the large haplotype-specific inversion on chromosome 8 could affect guide RNA specificity at the inversion breakpoints and could itself be exploited as a target for allele-selective editing. These diploid assemblies enable researchers to evaluate guide designs against the actual target genome, improving both the precision and reproducibility of genome editing experiments in these cell lines.

Several limitations should be noted. The assemblies were generated from sequencing data collected across multiple cell culture passages and therefore represent a composite of the genome across a window of the culture history rather than a single time point; passage-dependent variation accumulated before or after our sampling window is not captured. Although the assembly quality values exceed current community standards and we chose not to apply polishing at this time, future assembly polishing such as DeepPolisher [76] could modestly reduce remaining consensus errors. Similarly, tools such as NucFlag (https://github.com/logsdon-lab/NucFlag, last accessed 27 February 2026) could provide additional per-contig assembly quality assessment, particularly for repetitive regions such as centromeres. Finally, while we demonstrate improved read mapping and phasing using simple alignment-based approaches, we have not yet integrated these assemblies into a pangenome graph, which would more fully realize their potential for variant calling and haplotype-aware analysis.

The publicly available assemblies and variant calls from this study will serve as a valuable resource for the research community, facilitating improved rigor in studies using these model systems. The higher unique SV burden we observed in BJ and IMR-90 relative to HPRC samples (58%–59% versus 27% unique SVs) may in part reflect structural variation acquired during *in vitro* passaging, motivating future longitudinal studies comparing assemblies across different passage numbers. Cell-line-specific variants may also have tissue-of-origin relevance; for instance, variants unique to BJ could be pertinent to skin fibroblast biology while those in IMR-90 may relate to lung fibroblast function. This hypothesis could be tested by cross-referencing the variant calls with tissue-specific expression and eQTL databases. The phased assemblies also make allele-specific analyses immediately tractable, such as investigating escape from X-chromosome inactivation in 46,XX IMR-90 cells. More broadly, our work establishes a framework for diploid assembly of widely used *in vitro* model systems that could be extended to other foundational cell lines lacking complete haplotype-resolved references.

## Supplementary Material

gkag333_Supplemental_Files

## Data Availability

The HiFi, ONT-UL, Hi-C, and RNA-seq data generated in this study are available under NCBI BioProject accession number PRJNA1214805. The genome assemblies are available under NCBI BioProject accession numbers PRJNA1258368, PRJNA1258367, PRJNA1258366, and PRJNA1258365 for BJ hap1, BJ hap2, IMR-90 hap1, and IMR-90 hap2, respectively. The mitochondria genomes for BJ and IMR-90 are available at https://doi.org/10.6084/m9.figshare.31395558 (last accessed 27 February 2026) and https://doi.org/10.6084/m9.figshare.31395576 (last accessed 27 February 2026), respectively. The vcf file for SNVs and indels is available at https://doi.org/10.6084/m9.figshare.28924484 (last accessed 27 February 2026) and the vcf file for SVs is available at https://doi.org/10.6084/m9.figshare.28924511 (last accessed 27 February 2026). The HPRC Year 1 SV vcf file that we compared against can be downloaded from https://s3-us-west-2.amazonaws.com/human-pangenomics/pangenomes/freeze/freeze1/minigraph-cactus/hprc-v1.1-mc-chm13/hprc-v1.1-mc-chm13.vcfbub.a100k.wave.vcf.gz (last accessed 27 February 2026). The HPRC Release 2 assemblies can be downloaded from the URLs listed in https://github.com/human-pangenomics/hprc_intermediate_assembly/blob/main/data_tables/assemblies_release2_v1.0.index.csv (last accessed 27 February 2026). Publicly available Illumina data for BJ and IMR-90 were downloaded from the NCBI SRA database (BJ BioSamples ID: SAMD00260498, BioProject ID: PRJDB6632, Run ID: DRR258666; IMR-90 BioSamples ID: SAMN35312059, BioProject ID: PRJNA975175, Run ID: SRR24709136). Publicly available ChIP-seq data for BJ and IMR-90 were downloaded from ENCODE (BJ Experiment: ENCSR000DQH, Files: ENCFF001ERS and ENCFF001ESA; IMR-90 Experiment: ENCSR087PFU, Files: ENCFF591EQV and ENCFF742UXQ).
