# Supplementary material for: Haplotype-resolved genome assemblies of BJ and IMR-90 human fibroblast cell lines reveal extensive structural variation and enable reanalysis of historical sequencing data
Source: Nucleic Acids Res. 2026 Apr 29;54(8):gkag333. doi: 10.1093/nar/gkag333 (PMC13124242; doi:10.1093/nar/gkag333)
Supplement: gkag333_Supplemental_Files [file gkag333_supplemental_files.zip › BJ-IMR90-NAR_Supplementary Figures.docx]

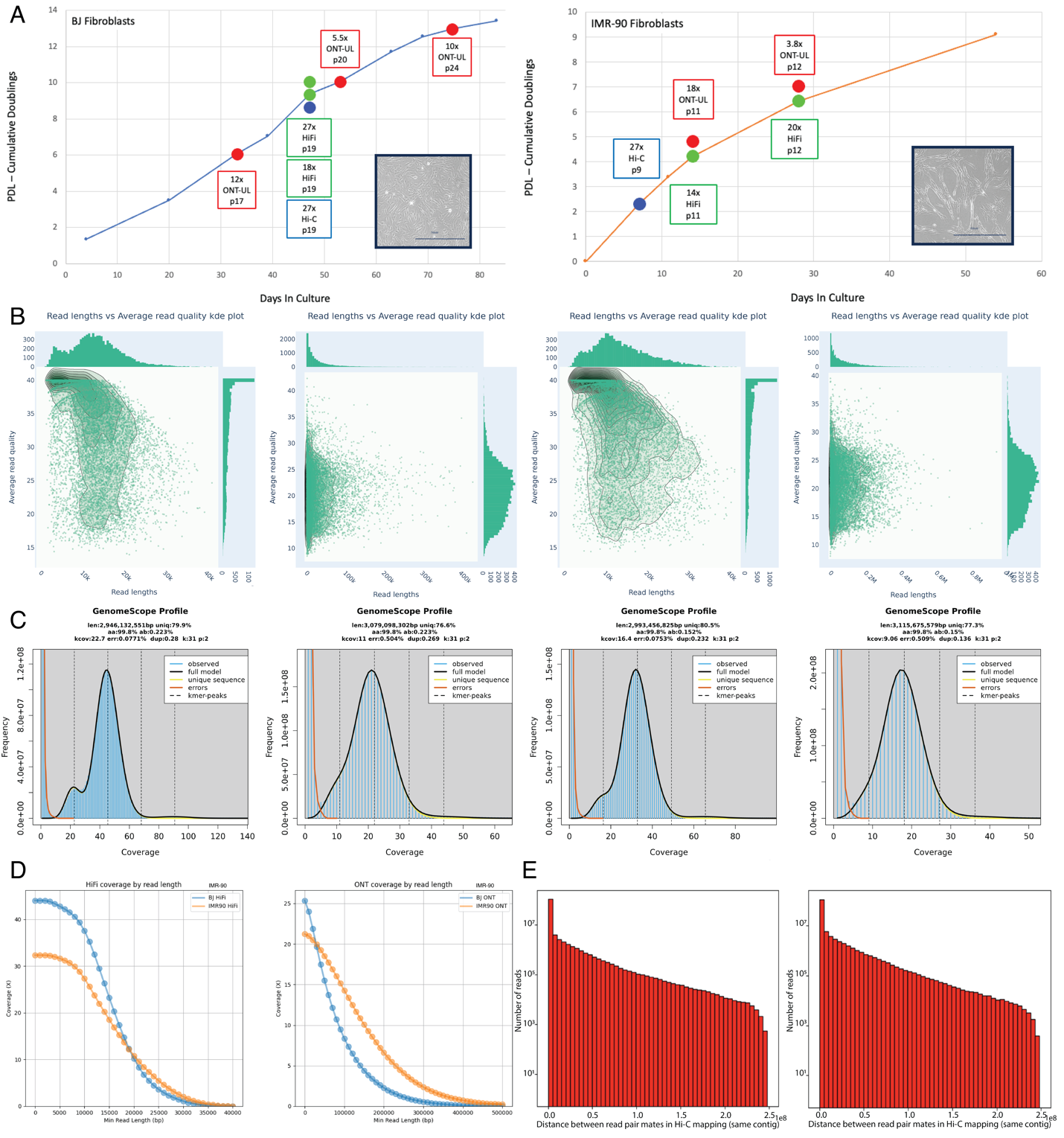


**Supplementary Figure 1:** **A** BJ and IMR-90 cell culture growth curves. Plots show cumulative doublings of cultured fibroblasts with time points where cells were harvested for different sequencing platforms (HiFi, Hi-C, ONT-UL, and RNA-seq). Passage number and sequencing coverage for each harvested time point are shown in boxes at each respective time point. Microscope images with representative examples of each cell type are shown in inset of each growth curve. **B** NanoPlot read length vs average read quality KDE plots for BJ HiFi, BJ ONT-UL, IMR-90 HiFi, IMR-90 ONT-UL data respectively. **C** GenomeScope plots for BJ HiFi, BJ ONT-UL, IMR-90 HiFi, and IMR-90 ONT-UL data respectively show estimated coverage and heterozygosity. **D** Coverage vs read length for BJ and IMR-90 for HiFi and ONT-UL data, respectively. **E** Hi-C QC results for BJ and IMR-90.


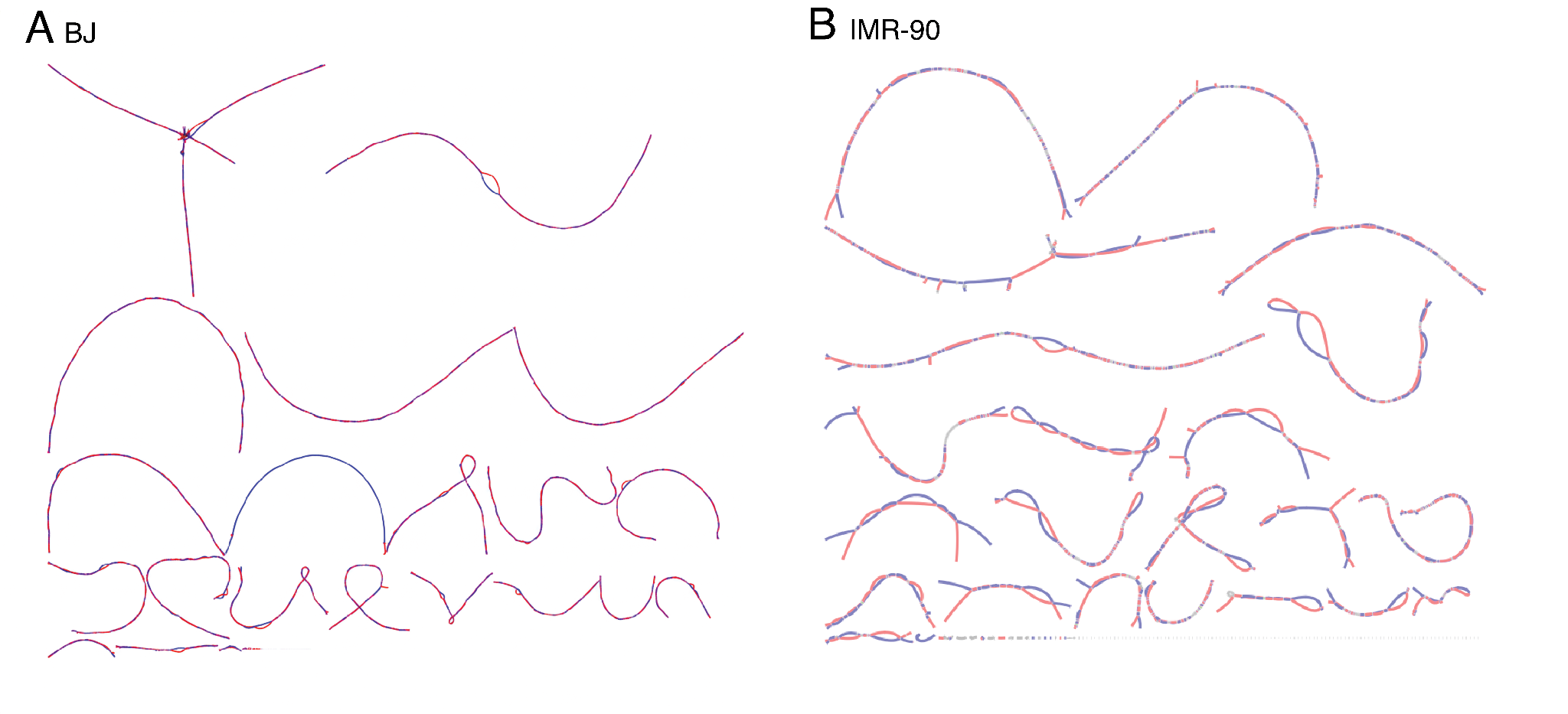


**Supplementary Figure 2:** Bandage plots of the Verkko assemblies for **A** BJ and **B** IMR-90. Most of the chromosomes are in a single contiguous piece. The acrocentric chromosomes also belong to the same piece as expected.


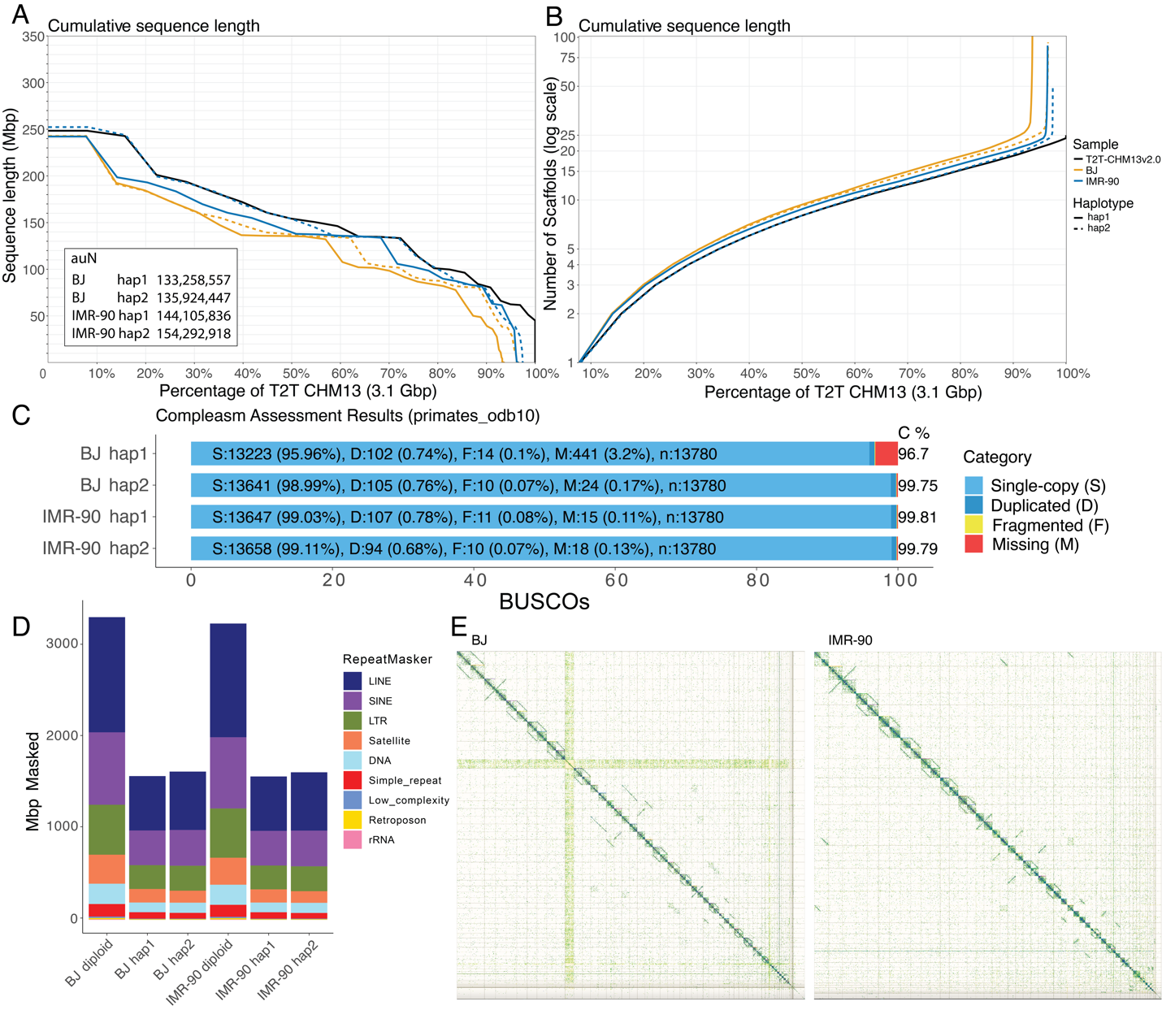


**Supplementary Figure 3:** Pre-curation assembly QC. **A** NGx chart showing the contiguity of the four pre-curation assembly haplotypes (based on the genome size of T2T-CHM13v2.0). The NG50 value (at x=50%) is the maximum sequence length such that all sequences of that length or longer together cover at least 50% of the reference genome. In black is the NGx plot for T2T-CHM13v2.0. Overlaid on the chart are the auN metrics for the pre-curation haplotypes. **B** LGx charts showing the number of contigs needed to reach a given percentage of the human genome. **C** The compleasm results showing the estimated number of single-copy (S), duplicated (D), fragmented (F), and missing (M) near-universal single-copy ortholog gene sequences in the pre-curation haplotypes. **D** RepeatMasker results showing the number of bases in the pre-curation haplotypes that are categorized into each repeat family. **E** Hi-C maps after mapping the Hi-C data to the pre-curation assemblies.


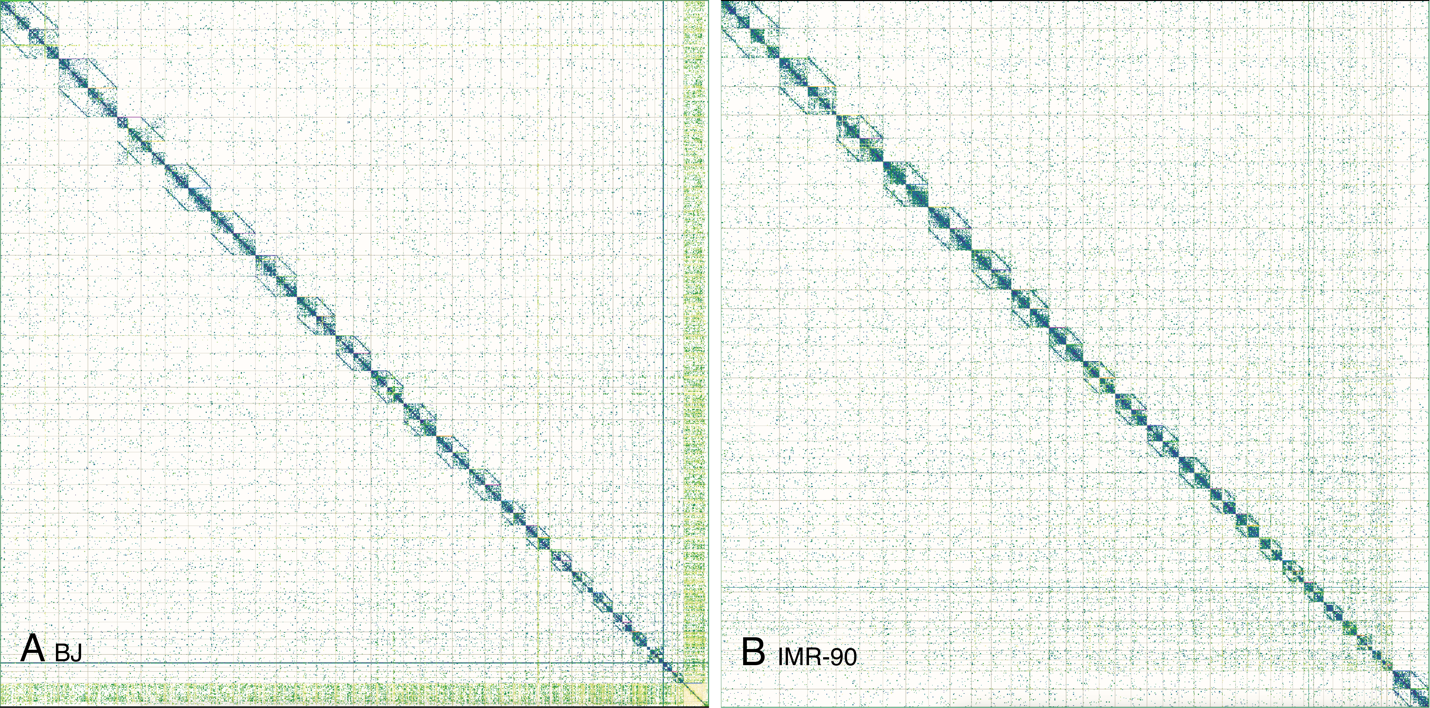


**Supplementary Figure 4:** Post-curation Hi-C maps for **A** BJ and **B** IMR-90. These Hi-C maps show the characteristic diamond shape for each diploid chromosome. There are no significant off diagonal contacts.


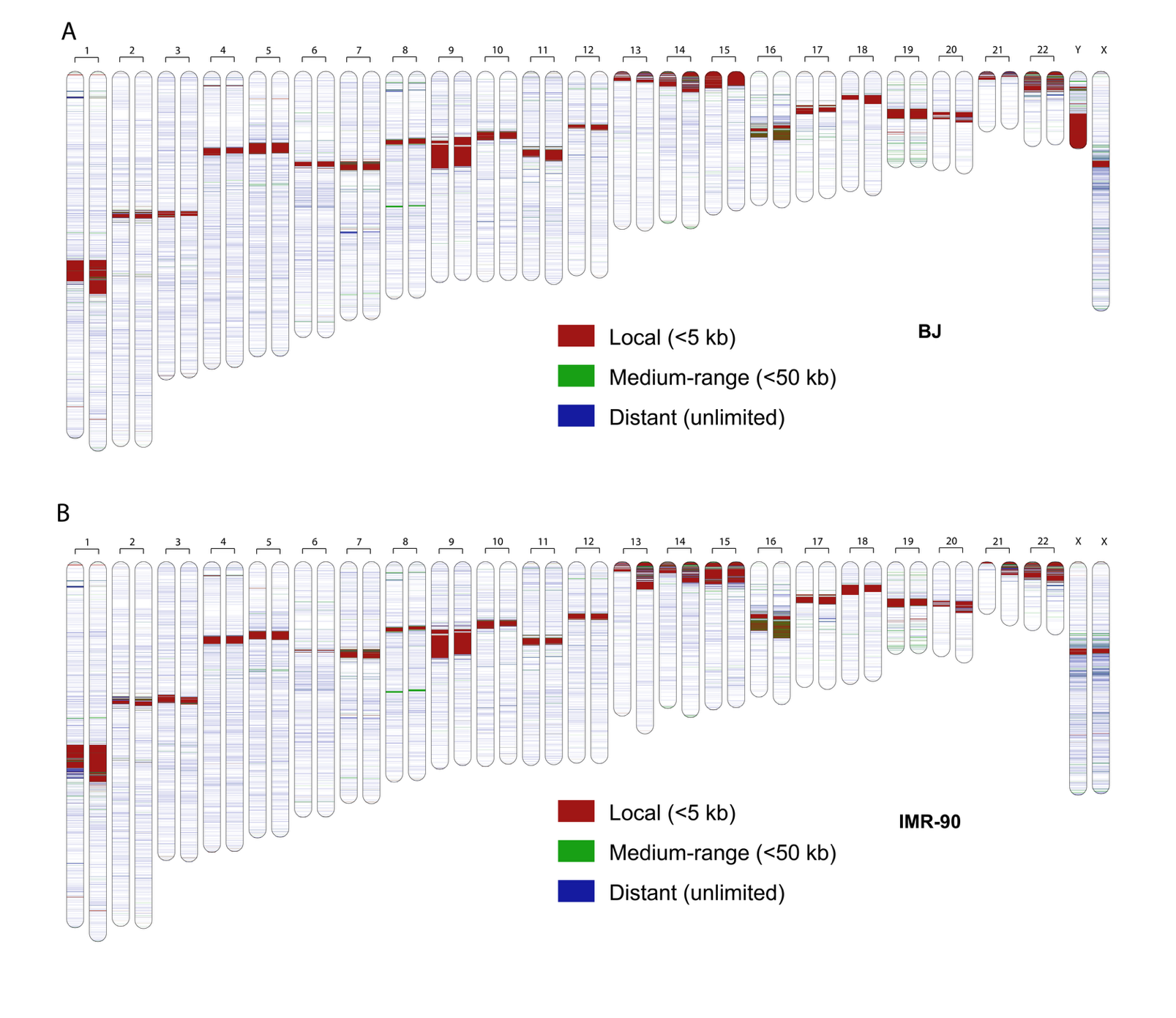


**Supplementary Figure 5:** AlcoR visualization of haplotype-resolved low-complexity regions in BJ and IMR-90 post-curation diploid assemblies. Genome-wide distribution of low-complexity regions (LCRs) identified by AlcoR across all chromosomes for **A** BJ and **B** IMR-90 cell lines. For each chromosome (1-22), the left and right bars represent haplotype 1 and haplotype 2, respectively. LCRs are colored by spatial distance class: red indicates local repeats (memory limit 5kb), green indicates medium-range repeats (memory limit 50kb), and blue indicates distant repeats (unlimited memory), corresponding to tandem/microsatellite repeats, regional duplications, and interspersed elements or segmental duplications, respectively. LRC regions were identified using bidirectional data compression with a detection threshold of 0.75, smoothing window of 5kb, and minimum region size of 5kb.


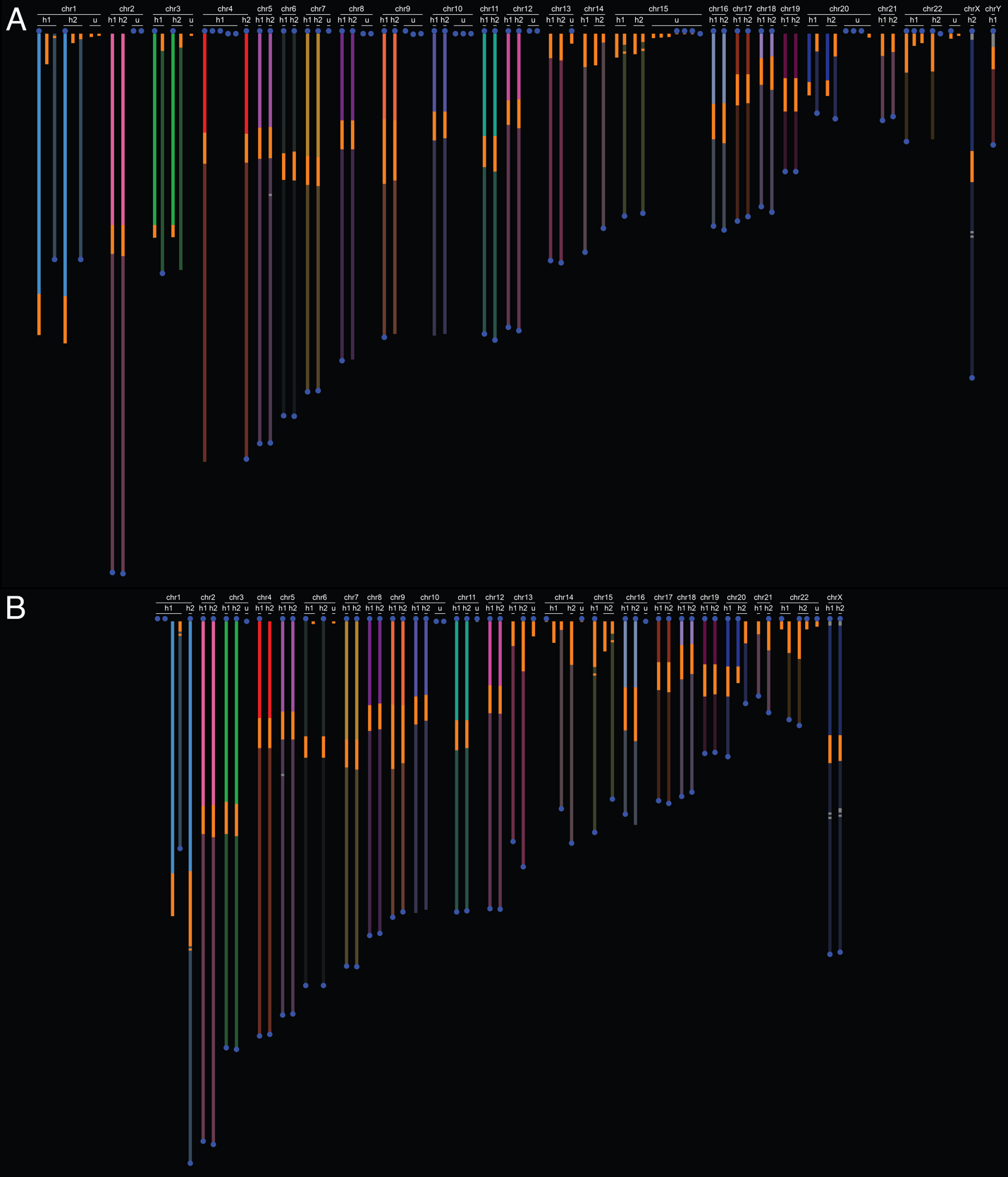


**Supplementary Figure 6****:** Computational karyograms for pre-curation **A** BJ and **B** IMR-90 assemblies (see **Methods**). Most of the chromosomes consist of a single contig. A few of the chromosomes consist of two contigs broken at the centromere. The sex chromosomes are assembled in a single contig with telomeres at each end.


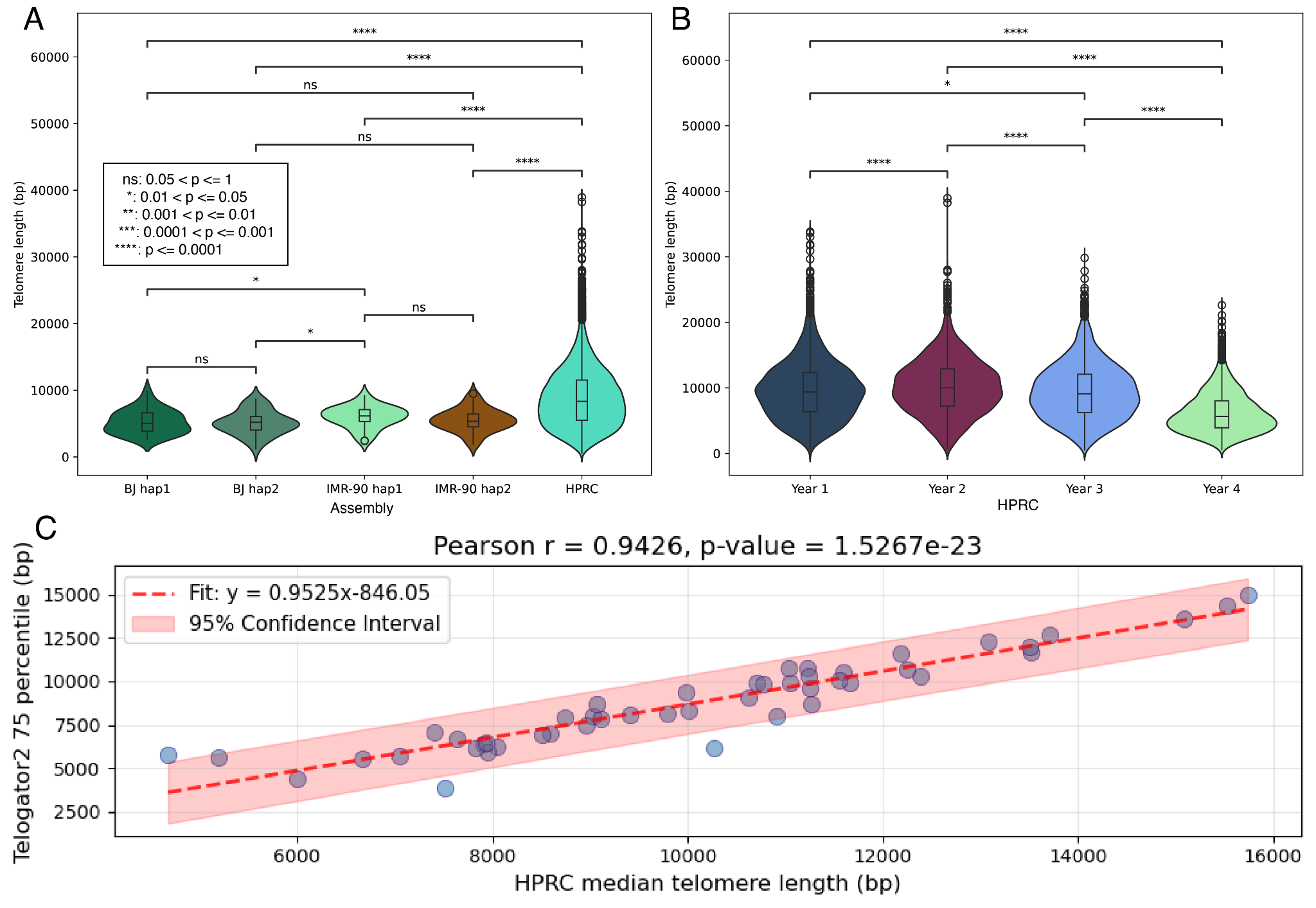


**Supplementary Figure 7**: **A** BJ and IMR-90 assembly-based telomere lengths compared to the HPRC human pangenome. Mann-Whitney-Wilcoxon two-sided tests were performed to compare the median telomere lengths. **B** Assembly-based telomere lengths in years 1, 2, 3 and 4 of the HPRC pangenome. **C.** Correlation across HPRC samples between per-sample 75th percentile read-based telomere lengths (across chromosome arms) estimated by telogator2 and per-sample median assembly-based telomere lengths (across chromosome arms) estimated by seqtk.


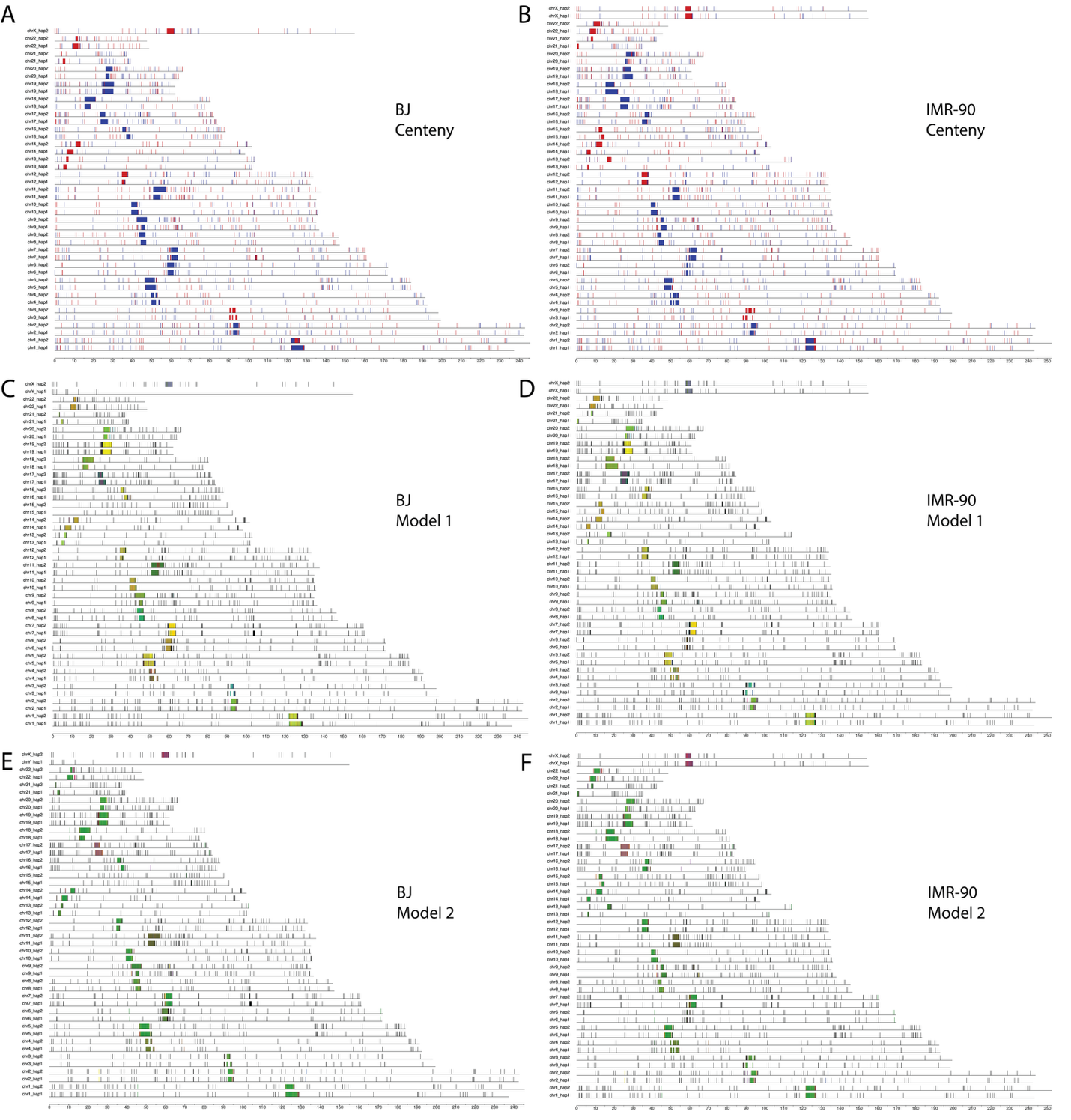


**Supplementary Figure 8**: Centeny maps and GCP models of BJ and IMR-90 assemblies. **A and B** Centeny maps of BJ and IMR-90 showing all CENP-B box positions at the centromere level and chromosome-wide, including Ecto Centromeric Sites (ECSs). The color indicates strand orientation: forward (+, red) and reverse (-, blue). **C and D** Genomic Centromere Profiling (GCP) Model 1 of BJ and IMR-90. **E and F** GCP Model 2 of BJ and IMR-90. Model 1 quantifies the base-pair distance between consecutive CENP-B boxes, while Model 2 captures the periodicity of those distances, corresponding to higher-order organizational patterns.


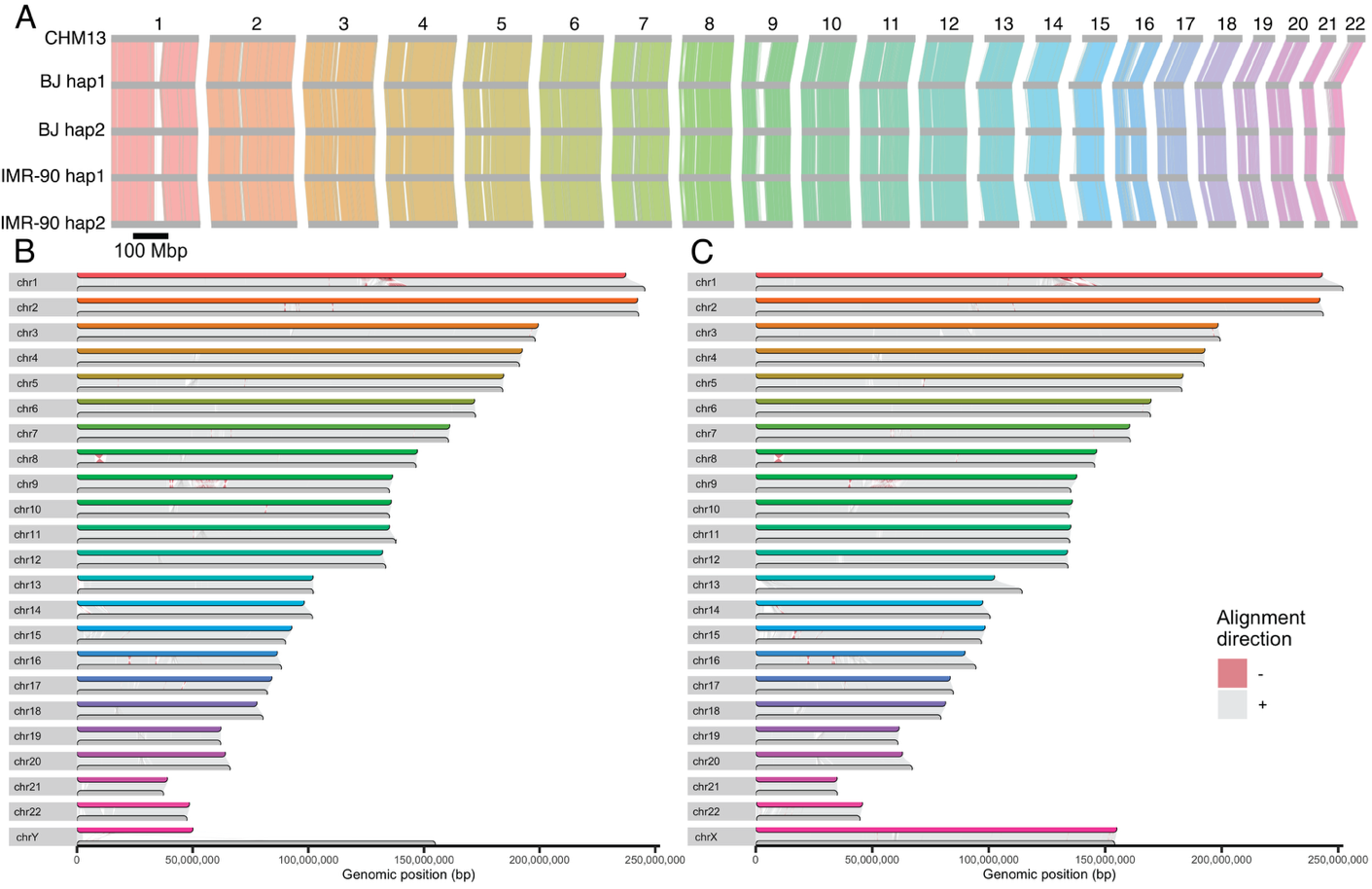


**Supplementary Figure 9****:** Post-curation assembly synteny plots. **A** Multi-sample synteny between T2T-CHM13v2.0 and the four post-curation assembly haplotypes generated by ntSynt. Sex chromosomes not shown. Conserved blocks are indicated by colored links between haplotypes. **B** and **C** Hap1 versus hap2 synteny plots for BJ and IMR-90 post-curation assemblies, respectively, generated by SVbyEye.

**
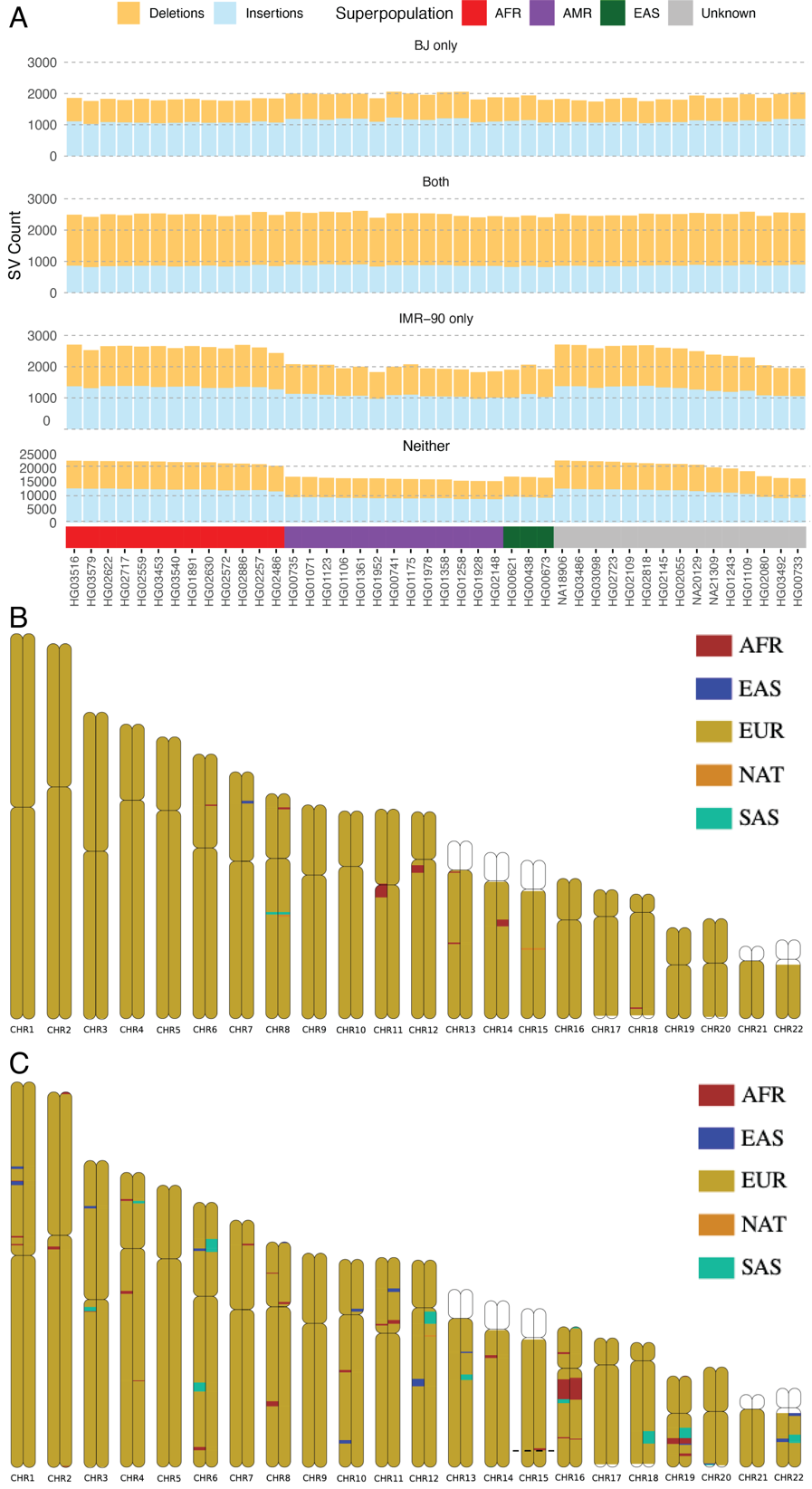
**

**Supplementary Figure 10**: **A** Comparison of SV insertions and deletions of HPRC pangenome to BJ and IMR-90. The pangenome samples are ordered by superpopulation. Pangenome variants are divided into four groups: “Both” if they are also present in both BJ and IMR-90, “BJ only” or “IMR-90” only if they are also present in that cell line but not the other, and “Neither” if they are not present in either cell line. The number of “BJ only” variants is highest for the AMR pangenome samples, while the number of “IMR-90” only variants is highest for AFR. **B** BJ chromosome painting based on ancestry. **C** IMR-90 chromosome painting based on ancestry.


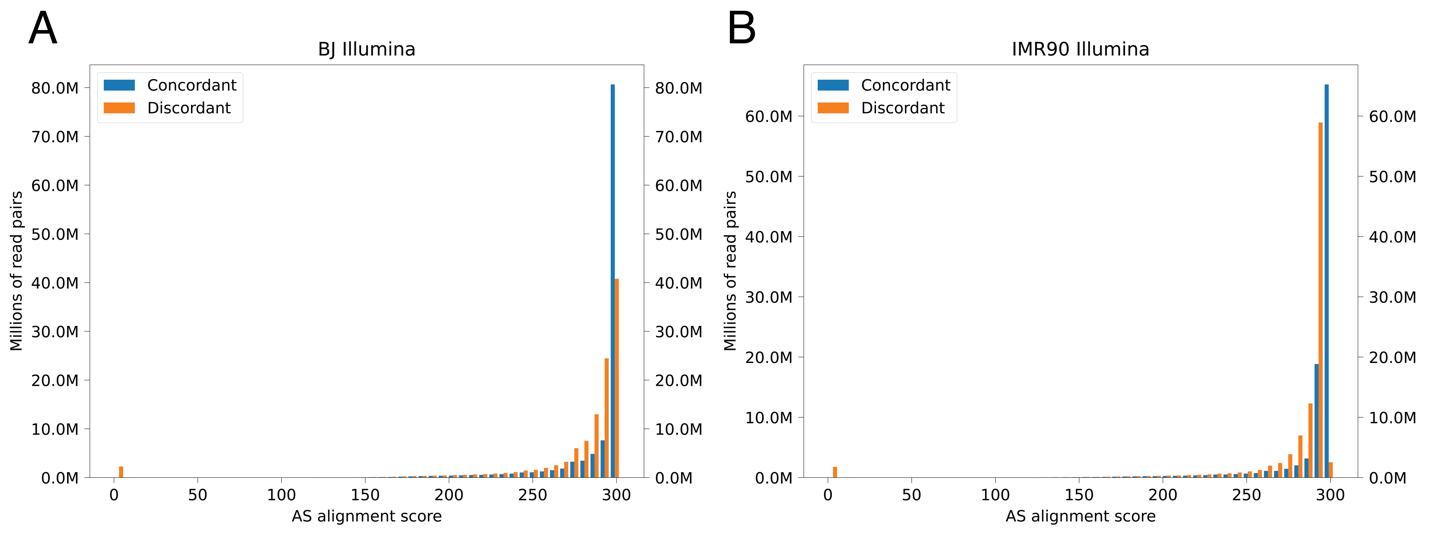


**Supplementary Figure 11****:** Summed BWA alignment score (AS) distributions of concordant versus discordant alignments of Illumina read pairs against the post-curation **A** BJ and **B** IMR-90 assemblies. The summed alignment scores range from 0 to 300 for each read pair, where 300 indicates that all 300 bp match the reference exactly. Deletions, insertions, and mismatches lower the score. Read pairs that are not properly mapped have a summed alignment score of 0. Concordant alignments (read pairs mapped to their AS-assigned haplotype) show higher alignment scores than discordant alignments (read pairs mapped to the opposite haplotype).
